# The Parkinson Disease‐Associated Mutant DNAJC13(N855S) Leads to Its Accelerated Degradation and Negatively Affects Macroautophagy and Retromer Complex‐Mediated Dynamics

**DOI:** 10.1002/jcp.70074

**Published:** 2025-07-27

**Authors:** Anna Stein, Stella Vo, Christian Freese, Joram Kluge, Joanna Maus, Ingrid Koziollek‐Drechsler, Beate Silva, Christian Behl, Albrecht M. Clement

**Affiliations:** ^1^ Institute of Pathobiochemistry University Medical Center of the Johannes Gutenberg‐University Mainz Germany; ^2^ Current Address: Department for Hematology, Cell Therapy and Hemostaseology University Hospital Leipzig Leipzig Germany

**Keywords:** autophagy, DNAJC13, endosomal traffic, endosome

## Abstract

While Parkinson′s disease has a multifactorial etiology, 5%–10% of cases present with identifiable disease‐causing gene mutations. Further investigation into these mutations is a way to identify underlying pathologic mechanism. One of the rare Parkinson‐associated genes is *DNAJC13*, coding for an endosome‐associated protein. Several lines of evidence suggest that disturbed endosomal pathways are instrumental in the development of Parkinson pathology. Recently, we have shown that DNAJC13/RME‐8 is a positive modulator of autophagy, a lysosome‐associated degradative process. Here, we further characterize the role of the disease‐linked DNAJC13(N855S) mutant and perform biochemical, cell biological, co‐localization, and expression analysis by employing a newly established cell line with reduced DNAJC13 expression and by transiently expressing the DNAJC13(N855S) mutant variant. We observed that the DNAJC13(N855S) variant is less stable than the wild‐type protein and might thus impact proteostasis. Furthermore, the protein has functional deficits as it cannot compensate for the impaired autophagic activity in cells with chronically reduced DNAJC13 levels. In addition, the DNAJC13(N855S) showed a dominant negative effect on the distribution of the cation‐independent mannose‐6‐phosphate receptor without affecting overall cathepsin D levels or activity. Lastly, we observed a decreased expression of several genes related to autophagy induction and biogenesis in stable DNAJC13 knockdown cells. Our data point toward a loss‐of‐function mechanism of the DNAJC13(N855S) variant and that chronically reduced DNAJC13 protein levels result in a reduced expression of genes largely involved in endosomal traffic and autophagosome biogenesis. The DNAJC13(N855S) mutant might thus cause disease in part by its instability and in part by a dominant negative effect on the autophagic pathway. These data support a pivotal role of endosomal pathway impairment in Parkinson′s disease pathogenesis.

## 1 Introduction

Parkinson′s disease (PD) is a neurodegenerative disease primarily characterized by its motor symptoms consisting of rigor, tremor, and bradykinesia. Pathologically, PD is defined by loss of dopaminergic neurons within the *substantia nigra* resulting in insufficient dopamine release. One of the pathological hallmarks of idiopathic and most genetic forms of the disease is the appearance of so‐called Lewy bodies. The prime constituent of these aggregates is α‐synuclein (α‐Syn). Most PD cases occur sporadically. Nonetheless, about 10% of all cases follow a familial inheritance pattern (de Lau and Breteler 2006) and more than 20 PD‐related genes have been identified so far (Blauwendraat et al. 2020).

**Figure 1:**
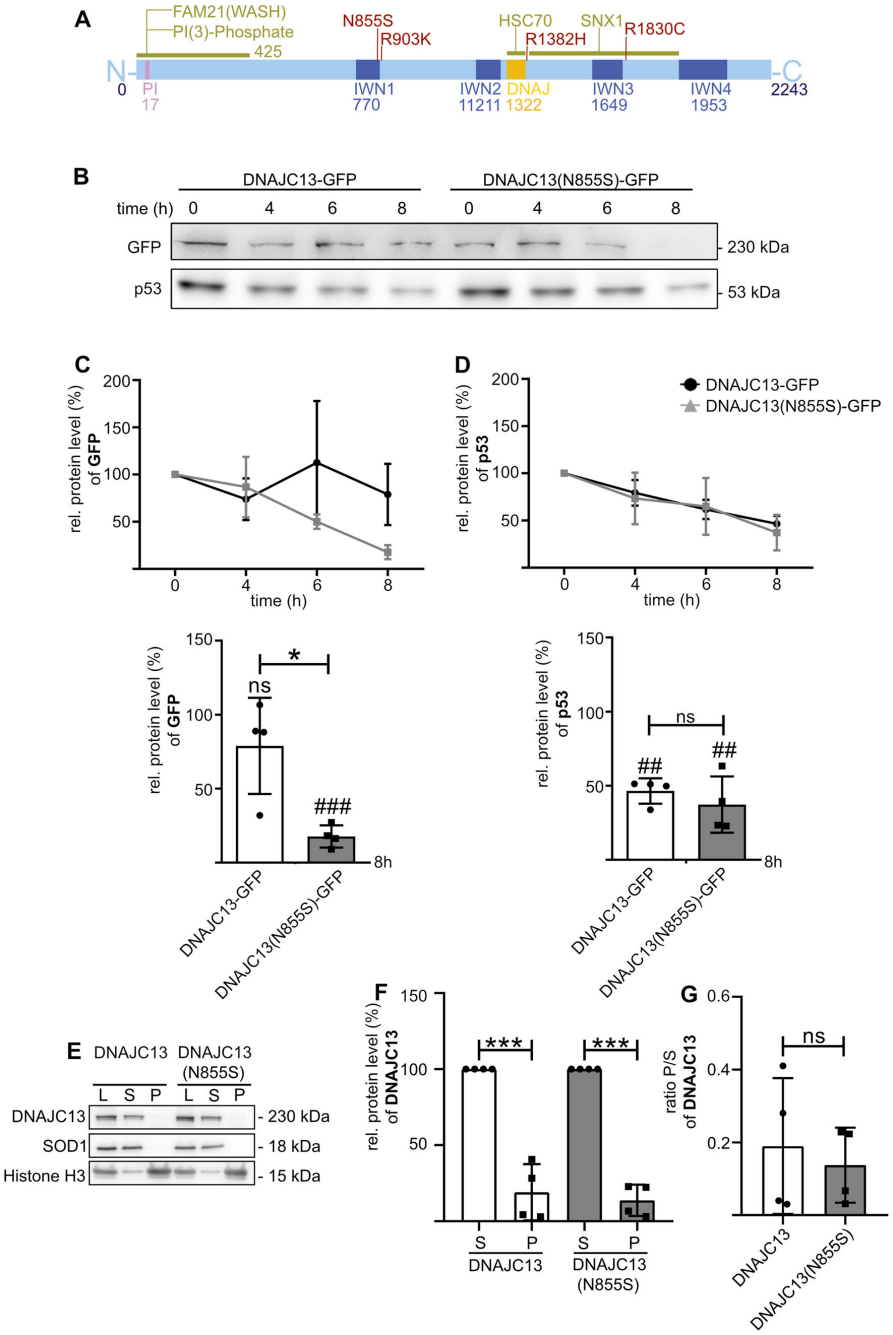
The DNAJC13(N855S) mutant protein is instable. (A) Schematic representation of the structure of human DNAJC13 including four identified PD‐associated mutations. The human protein consists of four IWN‐domains of repetitive amino acid sequences (isoleucine (I), tryptophan (W), and asparagine (N)). The central J‐domain enables the interaction with HSC70, whereas the very N‐terminal site interacts with PI(3)P for membrane association. The interaction site with FAM21 is located at the N‐terminus, while the interaction with SNX1 has been specified to a sequence between the J‐ and the IWN4‐domains. (B) HEK293T cells were transfected with GFP‐tagged DNAJC13 or DNAJC13(N855S). Twenty‐four hours after transfection, protein translation was inhibited by cycloheximide (4 µM) for indicated times. Equal amounts of protein were separated on an 8% SDS‐PAGE gel and after Western blotting, DNAJC13‐GFP and p53 were detected with specific antibodies directed against GFP and p53, respectively. Band intensities were quantified and normalized to protein levels at *t*_0_. The mean (±SD) out of four experiments is shown (C, D) DNAJC13‐GFP/(C) and p53 (D) protein levels were determined after 8 h treatment and compared to t_0_ (#) and between DNAJC13‐GFP groups (*) (mean ± SD; *n* = 4, *t*‐test: *p < 0.05; **p < 0.01; ***p < 0.001). (E) HEK293T cells were transfected with GFP‐tagged DNAJC13 or DNAJC13(N855S). Forty‐eight hours after transfection, the cells were lysed and total lysate (L) was divided into a soluble (S) and an aggregate‐enriched fraction (P) by centrifugation. Equal amounts of proteins were separated on an 8% SDS‐PAGE gel and protein was detected with specific antibodies after Western blotting. Endogenous superoxide dismutase 1 (SOD1) is a cytosolic protein characterizing the soluble fraction, histone H3 marks the aggregate‐enriched fraction. (F) Both DNAJC13 and DNAJC13(N855S) were barely detectable within the aggregate‐enriched fraction compared to supernatant. (G) The ratio of aggregate‐enriched (P) and soluble (S) fraction does not show any difference between DNAJC13 wild‐type and DNAJC13(N855S) mutant (mean ± SD; *n* = 4; *t*‐test: *p < 0.05; **p < 0.01; ***p < 0.001).

One of the rare genes associated with PD is *DNAJC13*. The DNAJC13(N855S) mutation was initially described in a Dutch‐German‐Russian‐Mennonite family (Vilariño‐Güell et al. 2014), although there are conflicting reports that mutations in the *TMEM230* gene caused PD in this pedigree (Deng et al. 2016). Nonetheless, several new mutations (DNAJC13 (R1382H) (Lin et al. 2019), DNAJC13(D1670G) (Li et al. 2020); DNAJC13(S1608R) (Gialluisi et al. 2021), DNAJC13(R903K) (Gagliardi et al. 2018), DNAJC13(R1830C) (Trinh et al. 2019) had been identified to cause PD in cohorts of different ethnicities in an autosomal‐dominant inheritance fashion. The *C. elegans* ortholog of human *DNAJC13* gene was initially identified as *receptor‐mediated endocytosis 8* (RME‐8) (Zhang et al. 2001) and a significant set of data was generated in the *C. elegans* model. Therefore, we use the term DNAJC13/RME‐8 where appropriate. Human *DNAJC13* encodes a protein of 2243 amino acids belonging to the group C of the heat shock protein 40 (HSP40)‐related protein family containing a so‐called J‐domain (Girard et al. 2005). Proteins with a J‐domain interact with heat shock cognate 70 (HSC70) and thereby largely function as co‐chaperons, as reviewed by Craig and Marszalek (2017). DNAJC13/RME‐8 carries the J‐domain in a central position flanked by two domains of unknown function to its N‐ as well as to its C‐terminal side, referred to as IWN‐domains (Figure 1A) (Girard et al. 2005; Zhang et al. 2001). The N‐terminus of the protein interacts with phosphatidylinositol‐3‐phosphate (PI(3)P), facilitating membrane association (Fujibayashi et al. 2008; Xhabija and Vacratsis 2015). DNAJC13/RME‐8 is mainly localized at the early endosome (Girard et al. 2005; Girard and McPherson 2008; Gomez‐Lamarca et al. 2015; Popoff et al. 2009; Shi et al. 2009; Zhang et al. 2001) and physically interacts with sorting nexin 1 (SNX1) (Popoff et al. 2009; Shi et al. 2009) and FAM21 (Freeman et al. 2014) that belong to the retromer and the WASH complex (Wiskott–Aldrich syndrome homolog), respectively. Although the function of DNAJC13/RME‐8 is not fully understood, several lines of evidence suggest that DNAJC13/RME‐8 is a key factor in organizing microdomains within the early endosome (Norris et al. 2017; Popoff et al. 2009; Shi et al. 2009), thus mediating protein traffic to the recycling or the degradative pathway (Freeman et al. 2014; Norris et al. 2017). Moreover, DNAJC13/RME‐8 is a positive modulator of autophagy (Besemer et al. 2021; Swords et al. 2023). One line of evidence suggests that DNAJC13/RME‐8 is required for autophagic lysosome reformation (Swords et al. 2023), whereby another line of evidence demonstrates that DNAJC13/RME‐8 affects the traffic of ATG9A from endosomal compartments toward the phagophore (Besemer et al. 2021). Interestingly, overexpression of wild‐type DNAJC13, but not the PD‐linked DNAJC13(N855S) mutant, enhanced autophagic flux (Besemer et al. 2021).

The retromer complex consists of two major subcomplexes, the heterotrimeric part containing vacuolar protein sorting (VPS) 26, VPS35, and VPS29 acting as cargo selection complex and the dimeric complex made up of varying SNX‐BAR proteins (Cui et al. 2018; Seaman 2021). The complex regulates retrograde transport routes toward the Golgi apparatus or the cell surface (Seaman 2021). Evidence indicates that the cargo selection is not exclusively mediated by the VPS cargo selection complex but also, at least for some cargoes like the cation‐independent mannose‐6‐phosphate receptor (CI‐MPR), by the SNX dimer (Kvainickas et al. 2017; Simonetti et al. 2017). The SNX proteins induce membrane curvature, which results in membrane tubulation and subsequently in budding vesicles (Harrison et al. 2014; Kovtun et al. 2018; Lucas et al. 2016). In this context, DNAJC13/RME‐8 coordinates the activity of the WASH complex by the interaction with the FAM21 tail (Freeman et al. 2014). The WASH complex is a protein pentamer mediating actin filament network formation and thus membrane dynamics. As increased tubulation upon lack of DNAJC13/RME‐8 was described early on (Popoff et al. 2009), mechanistic insight came from the study on the physical interaction of DNAJC13/RME‐8 with FAM21 and SNX1, (Freeman et al. 2014) showing the importance of DNAJC13/RME‐8 in WASH regulated membrane tubulation. As a retromer regulator, DNAJC13/RME‐8 also influences the trafficking of epidermal growth factor receptor (EGFR) and the transferrin receptor (Tf‐R) (Fujibayashi et al. 2008; Girard and McPherson 2008). Interestingly, the overexpression of DNAJC13(N855S) seems to impair the transport of EGFR from the early endosome to the late endosome as well as the trafficking of Tf‐R (Vilariño‐Güell et al. 2013; Yoshida et al. 2018). This seems to interfere with α‐Syn homeostasis, since the incubation of α‐Syn on DNAJC13(N855S) mutated cells displayed an accumulation of α‐Syn within the early endosomal compartment (Yoshida et al. 2018).

Here, we characterized the properties of the PD‐associated DNAJC13(N855S) variant and analyzed the stability of the protein. Furthermore, we investigated whether mutant DNAJC13(N855S) interferes with the subcellular localization of the CI‐MPR and cathepsin D activity. We found that upon mutant DNAJC13(N855S) expression, CI‐MPR is distributed more dispersedly within the cell, while not affecting its co‐localization with the trans‐Golgi network. In contrast, the mutant protein showed a reduced co‐localization with CI‐MPR compared to DNAJC13(WT). Interestingly, this does neither affect overall cathepsin D levels nor its activity within lysosome‐enriched fractions. Moreover, expression data show that genes responsible for autophagosome membrane integrity and related to ATG9A trafficking are downregulated in stable *DNAJC13* knockdown cell lines and that the DNAJC13(N855S) mutant cannot restore autophagic activity in these cells, supporting the view that DNAJC13/RME‐8 plays an important role in autophagy. This might contribute to the pathomechanism in DNAJC13‐mediated PD.

## 2 Materials and Methods

### 2.1 Cell Culture

All experiments were performed in human embryonic kidney cell 293 (HEK293) line variants HEK‐T and HEK‐A. Cells were cultured in Dulbecco's modified Eagle's medium with high glucose (DMEM, Life Technologies, 41965062) supplemented with 10% fetal calf serum (PAA Laboratories, A15‐101), 1% sodium pyruvate (Life Technologies, 1136‐088), and a mixture of antibiotics and antimycotics (Life Technologies, 15240‐112) in a humified atmosphere (37°C, 5% CO_2_). To establish stable *DNAJC13*‐knockdown lines, HEK293‐T were transfected by the calcium phosphate precipitation method as described previously (Liebl et al. 2014), using shRNA plasmid (#TRCN0000243950) from the TRC‐library (Sigma‐Aldrich). Subsequently, cells were selected under puromycin (2 ng/µL; Sigma‐Aldrich, P8833) treatment and individual colonies were picked. Clones with the most efficient knockdown were selected.

For transient overexpression of DNAJC13(WT) or DNAJC13(N855S), cells were transfected by the calcium phosphate precipitation method. Expression plasmids were generated and sequenced as described previously (Besemer et al. 2021). Twenty‐four hours after transfection, cultures were washed and 48 h after transfection cells were used for experiments. To block lysosomal acidification, cells were incubated with 4 µM bafilomycin A_1_ (LC Laboratories, B‐1080) for 4 h. Since bafilomycin A_1_ is soluble in DMSO, controls were incubated in corresponding concentrations of DMSO (Roth, A994.2). To determine the half‐life of DNAJC13, transiently transfected cells were incubated with the translation inhibitor cycloheximide (4 µM, Calbiochem, 239764) for the times indicated.

### 2.2 Western Blot

For protein analysis, cells were harvested in lysis buffer (62.5 mM Tris–HCl, 2% [w/v] SDS and 10% sucrose [pH 8], EDTA‐free protease [Roche, 04693159001] and phosphatase inhibitor cocktails [Roche, 04906837001]). Lysates were sonicated twice with a tip sonicator at 60 Hz for 10 s on ice. Lysates were directly analyzed or stored at −20°C over night. Equal amounts of proteins were separated on 8% or 15% SDS‐PAGE gels and subsequently transferred onto a nitrocellulose membrane. Before antibody incubation, membranes were blocked with 4% (w/v) fat‐free milk powder (Applichem, A0830) in phosphate‐buffered saline supplemented with 0.05% TWEEN20 (PBS‐T). Proteins were detected by specific antibodies: SQSTM1 (Progen, GP62‐C), LC3B (Novus, NB‐100‐2220), FLAG (Sigma, F1804), tubulin (Sigma, T9026), SOD1 (Epitomics, 2018‐1), ATG9A (Abcam, ab108338), LAMP1 (Abcam, ab24170), LAMP2A (Abcam, ab18528), cathepsin D (Abcam, ab75852), p53 (Cell Signaling, 2524). A polyclonal antibody directed against DNAJC13 was produced by Proteintech (Martinsried, Germany) in rabbits immunized with the N‐terminal peptide RGKYKRVFSV. Subsequently, species‐specific and horseradish peroxidase‐coupled secondary antibodies were applied (anti‐rabbit, Jackson Immuno Research, 711‐035‐152; anti‐mouse, Jackson Immuno Research, 715‐035‐151; anti‐guinea pig, Jackson Immuno Research, 706‐035‐148). Enhanced luminescence signals were detected (Amersham Imager 600, GE) and ImageJ was used for densitometric quantification.

### 2.3 Immunocytochemistry

Cells were either grown on glass coverslips or Ibidi imaging chambers, fixed with 4% (w/v) paraformaldehyde (Sigma, P6148), and permeabilized with methanol (−20°C, 95%, 6 min), following several washing steps with PBS (Sigma, D8537). Coverslips were incubated with specific primary antibodies: cation‐independent mannose‐6‐phosphate receptor (CI‐M6PR) (Abcam, ab8093), trans‐Golgi network protein 2 (TGN46) (ABD Serotec, AHP500), early endosome A1 (EEA1) (BD Biosciences, 610457), sorting nexin 1 (SNX1) (BD Biosciences, 611482), transferrin‐receptor (Tf‐R) (Invitrogen, 13‐6800), vacuolar protein sorting 35 (VPS35) (LSBio, LSB5909/55567), WAS protein family homolog 1 (WASH) (millipore, ABS72). All of them were washed with PBS and species‐specific secondary antibodies were applied (anti‐mouse‐Cy5 [Jackson Immuno Research, 715‐175‐150], anti‐sheep‐sheep‐Cy3 [Jackson Immuno Research, 713‐165‐147]). DAPI (Calbiochem, 382061) was used to detect nuclei. The coverslips were mounted and imaged with a Zeiss 710 confocal microscope. The unprocessed images were analyzed by ImageJ through defining the positively transfected cell as the region of interest and then calculating Pearson´s correlation (r) of two fluorescence signals, using the Coloc2‐tool. See figure legends for details related to the number of biological replica and statistical analysis.

### 2.4 Enzyme Activity Assay of Cathepsins

The activity of cathepsins was determined according to a modified protocol established by Gumpper et al. (Gumpper et al. 2019). In brief, after harvesting, cells were lysed in the three‐fold amount (w/v) of a cytosolic low salt buffer to preserve lysosomal integrity (20 mM Tris‐HCl, 1 mM EDTA, 1 mM EGTA, 1% glycerol, 2 mM DTT, pH 7.8). Subsequently, the suspension was centrifuged for 30 min (4°C, 13,000 *g*). To break the lysosomal membrane, the pellet was resuspended using double amount (w/v) of acid buffer (200 mM sodium‐acetate, 50 mM NaCl, 0,1% TritonX‐100, pH 5.0). The suspension was sonicated twice for 10 s at 60 Hz, following another centrifugation (30 min, 4°C, 13,000 *g*). The supernatant was used for the determination of lysosomal enzyme activity. The enzyme activity of equal amounts of protein was determined by adding a fluorogenic substrate and measuring the fluorescent signal over time. Therefore, 1 µg of protein was diluted into enzyme activity buffer (100 mM sodium acetate, 120 mM NaCl, 1 mM EDTA, pH 5.5) to a total volume of 50 µL in a light‐protected microtiter plate. For negative control, pepstatin A (0.1 mM; Merck, 516481), an inhibitor for cathepsin D and other aspartyl proteases, was added. The plate was incubated for 15 min at 37°C for equilibration. Subsequently, 50 µL of cathepsin D/E substrate (20 µM; Enzo, BML‐P145) diluted in activity buffer was added and measurement was started immediately (Varioscan Lux, ThermoFischer). Fluorogenic signals were recorded every 30 s for a total time of 1 h (excitation 340 nm, emission 420 nm). For quantification, the slope of the curve in the linear range was determined. In a different set of experiments, the activity of other cathepsins was determined as above with some modifications: 7.5 µg of protein from lysosome‐enriched preparations was diluted in enzyme activity buffer to a total volume of 100 µL in a light‐protected microtiter plate. For negative control, the cysteine protease inhibitor (including cathepsin B and L) E‐64‐D (final concentration 0.5 mM) was used. Fifty microliters of fluorogenic substrate (40 µM benzyloxycarbonyl‐Phe‐Arg‐[7‐amino‐4‐methylcoumarine], Enzo Life Sciences, BML‐P139) was added to each well and fluorogenic signals were recorded after equilibration for 5 min at 37°C every 3 min with the Fluoroskan Ascent FL (Thermo Labsystem) (excitation at 355 nm, emission at 460 nm).

### 2.5 Cell Fractionation Assay

The method was performed as described earlier (Witan et al. 2008) In brief, HEK293T cells were transiently transfected with GFP‐tagged DNAJC13 constructs by the calcium‐phosphate method. After 48 h, cells were extracted in PBS supplemented with proteinase inhibitor cocktail (Roche, 04693159001) and sonicated. Lysates were separated by centrifugation (17,000 *g*, 4°C, 15 min). The supernatant was collected and the pellet was washed one time. The final pellet was resuspended in SDS‐lysis buffer. Total lysate, supernatant and pellet were separated on SDS‐PAGE and analyzed by Western blot.

### 2.6 RNA‐Quantification

To determine expression levels, mRNA was quantified by RT‐qPCR. For RNA‐extraction, the RNA‐Miniprep Kit (Agilent Technologies, 400800) was used. Concentration of nucleic acids was determined by Nanodrop1000 (Peqlab). For the conversion into cDNA the Omniskript RT Kit (Qiagen, 205113) was used according to the manufacturer′s protocol. Following the SensiFAST SYBR & Fluorescein Kit (Bioline, Bio‐96020) protocol, the PCR reaction was run with the iQ Real‐Time‐PCR Thermocycler (BioRad). DNMT1 was considered as the housekeeping gene. All primers used are listed in Table S1. Cycle‐threshold results were calculated about DNMT1 using the REST2009 software (Pfaffl et al. 2002).

### 2.7 Statistical Analysis

For results of all experiments determining protein levels variances were calculated, following Student´s *t*‐test in case of comparison of two groups and one‐way ANOVA to compare several groups. In case of one‐way ANOVA, Bonferroni post hoc correction was applied. The immunocytochemistry results were analyzed by ImageJ. After determining a region of interest, Pearson´s correlation was calculated. For comparison of two conditions, Pearson´s correlation for each condition was compared using Student´s *t*‐test. All statistical calculations were performed using GraphPad Prism 5. Statistics are detailed with legends to every figure.

## 3 Results

### 3.1 The DNAJC13(N855S) Is Less Stable Than DNAJC13(WT)

As a molecular basis for mutant DNAJC13(N855S) toxicity, a loss of function, a gain of function, dominant negative effects, or an unstable protein could be postulated. To test the latter, we investigated if DNAJC13(N855S) is less stable compared to the wild‐type protein. Cells overexpressing GFP‐tagged DNAJC13 were treated with cycloheximide to block protein synthesis for 8 h. Subsequently, protein levels of DNAJC13‐GFP were determined by Western blot (Figure 1B,C) whereby p53 (Figure 1B,D) and HSP90 (Figure S1) served as controls for short‐lived and stable proteins, respectively. P53 is degraded via the ubiquitin‐proteasome pathway. After cycloheximide treatment significantly less mutant protein was detected compared to wild‐type DNAJC13 (Figure 1C), suggesting that the DNAJC13(N855S) is less stable compared to the wild‐type protein. Interestingly, degradation of p53 was independent of DNAJC13 variant overexpression (Figure 1D), indicating that the mutant DNAJC13(N855S) overexpression did not affect proteasomal degradation.

Accelerated degradation of the mutant DNAJC13 variant points toward an instable protein that might result in the formation of protein aggregates. To analyze this in more detail, we performed a centrifugation‐based aggregation assay as described earlier (Witan et al. 2008). Histone H3 and SOD1 served as positive control for the aggregate‐enriched and the soluble fractions, respectively. Both DNAJC13 variants were barely detectable within the aggregate‐enriched pellet (Figure 1E,F). The ratio of pellet/supernatant did not show any significant difference between wild‐type and mutant protein (Figure 1G), indicating no significant aggregation due to the point mutation.

### 3.2 DNAJC13(N855S) Impairs Autophagy

**Figure 2:**
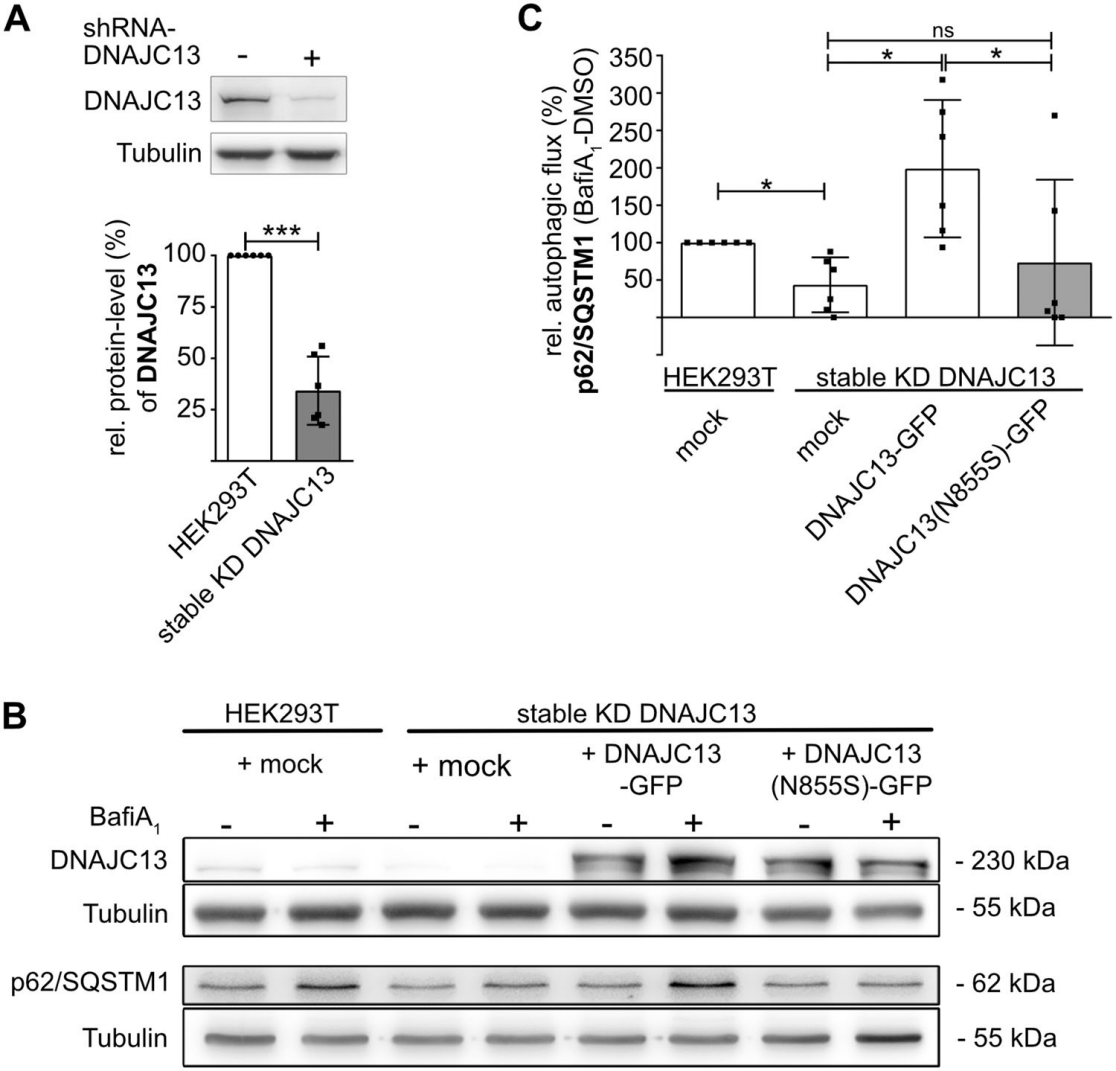
DNAJC13(N855S) cannot restore autophagic activity. (A) DNAJC13 protein levels of HEK293T cells stably transfected with a shRNA‐construct directed against the 3′‐untranslated region of *DNAJC13* were determined by Western blot. Tubulin served as loading control (mean ± SD; *n* = 6; *t*‐test, ***p < 0.001). (B) and (C) Naïve HEK293T cells or HEK293T cells stably expressing *DNAJC13* shRNA were transfected with empty vector (mock), GFP‐tagged DNAJC13 or DNAJC13(N855S). Forty‐eight hours after transfection, cells were treated with bafilomycine A_1_ (BafiA_1_: 4 µM) or DMSO for 4 h. (B) Equal protein amounts of total lysates were separated by SDS‐PAGE and protein levels of DNAJC13, tubulin, and p62/SQSTM1 were determined by Western blot. (C) Autophagic flux was determined by subtracting the signal intensities of DMSO‐treated cells from bafilomycine A_1_‐treated cells and represented normalized to naïve cells (mean ± SD; *n* = 6; one‐way‐ANOVA, Bonferroni post hoc analysis: *p < 0.05; **p < 0.01; ***p < 0.001).

We previously demonstrated that transient overexpression of DNAJC13 in naïve cells, but not its mutant DNAJC13(N855S), increased autophagic flux (Besemer et al. 2021). Furthermore, transiently reduced DNAJC13 protein levels resulted in an altered trafficking of ATG9A concomitant with a decreased autophagic activity (Besemer et al. 2021). To test, whether a stable knockdown of DNAJC13 has a comparable effect or whether compensatory mechanisms are activated, we generated HEK293T cells expressing shRNA directed against the 3´‐untranslated region (UTR) of the *DNAJC13* gene. These cells displayed a consistent reduction of about 70% of DNAJC13 protein levels compared to untransfected controls (Figure 2A). To determine whether autophagic flux is altered upon chronically reduced DNAJC13 protein levels, cells were treated with bafilomycin A_1_ and the protein level of autophagic cargo (here p62/SQSTM1 for sequestosome 1) was analyzed. Bafilomycin A_1_ blocks the V‐ATPase and thus the acidification of lysosomes, which, in turn, results in a reduced fusion of lysosomes with autophagosomes and impaired degradation of autophagic cargo. Therefore, the accumulation of autophagic cargo is a proxy for the generation of autophagosomes and autophagic flux. In line with the data from transient knockdown of DNAJC13, the stable HEK293T line showed a reduced autophagic activity (Figure 2B,C), indicating that the DNAJC13‐mediated reduction of autophagic activity is tolerated under steady‐state conditions. To investigate, whether the re‐expression of DNAJC13 can compensate the reduced autophagic activity, knockdown cells were transiently transfected with plasmids harboring *DNAJC13* or its mutant variant *DNAJC13(N855S)* cDNA, resulting in the expression of comparable DNAJC13 protein levels (Figure S2A). Indeed, after re‐expression of DNAJC13, autophagic activity was increased (Figure 2B,C). In contrast, upon re‐expression of DNAJC13(N855S) autophagic flux showed no statistically significant increase and remained at a low level in knockdown cells. These data are indicating that DNAJC13(N855S) represents a loss‐of‐function mutation with respect to its modulatory capacity in autophagosome biogenesis. It is of note, that DNAJC13(WT) as well as the mutant variant did not accumulate in autophagic vesicles (Figure S2B–D), confirming our previous results in a transient knockdown system (Besemer et al. 2021).

### 3.3 Expression of DNAJC13(N855S) Alters CI‐MPR Trafficking

**Figure 3:**
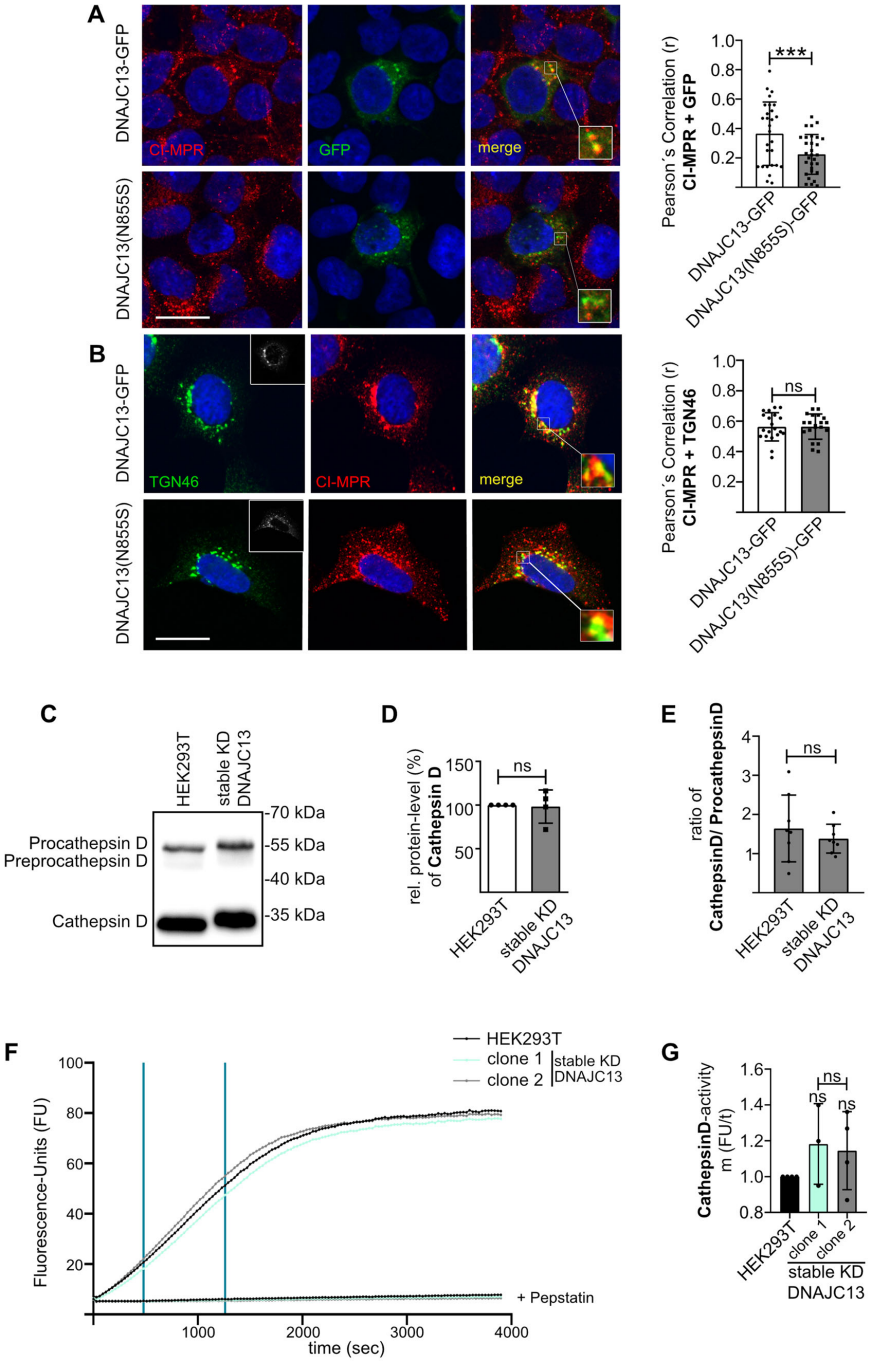
Overexpression of DNAJC13 (N855S) alters the subcellular co‐localization of CI‐MPR with DNAJC13, without affecting the cathepsin D processing or activity. HEK293A cells were transfected with GFP‐tagged DNAJC13 variants. After 48 h, cultures were fixed and co‐stained with CI‐MPR (A) or CI‐MPR and the trans‐Golgi network marker TGN46 (B) with appropriate fluorescent secondary antibodies. Co‐localization of GFP with CI‐MPR (A) and TGN46 with CI‐MPR was analyzed on confocal images and Pearson′s correlation was determined. Only transfected cells were considered (see box in (B), left panels, showing GFP‐positive cells) (mean ± SD; *n* = 30 individual cells from at least three independent experiments; *t*‐test: *p < 0.05; **p < 0.01; ***p < 0.001) (scale bar: 20 µm). (C) Lysosome‐enriched fractions were characterized by Western‐Blot analysis showing cathepsin D and its precursor proteins pro‐cathepsin D and prepro‐cathepsin D. (D) Quantification of the Western blot in C: Cathepsin D protein levels in stable DNAJC13‐knockdown cells were normalized to untransfected cells (mean ± SD; *n* = 4, *t*‐test: *p < 0.05; ns not significant). (E) The ratio of cathepsin D and pro‐cathepsin D is unchanged despite knockdown of DNAJC13 (mean ± SD; *n* = 4, *t*‐test: *p < 0.05; ns not significant) (F/G). Lysosome‐enriched fractions of wild‐type cells and two independent DNAJC13 knockdown clones were incubated with a specific fluorogenic substrate for cathepsin D/E. The linear increase of fluorescence over time (slope of the graph within lines) is a measure for the activity of the enzyme. (F) Shown is one representative experiment with technical triplicates. Pepstatin A is a specific inhibitor for cathepsin D and other aspartyl proteases and serves as negative control. (G) Each experiment was performed with technical triplicates. The slopes of mutant cells were normalized to controls. The enzymatic activity of cathepsin is independent of DNAJC13 protein levels. (mean ± SD; *n* = 4, *t*‐test: *p < 0.05; ns not significant).

DNAJC13 is functionally linked to the retromer complex through its direct interaction with SNX1 (Figure 1A) (Popoff et al. 2009; Shi et al. 2009). Substantial evidence suggests that the retromer complex plays a prominent role in PD pathogenesis, as mutations in VPS35, a core component of the complex, cause familial forms of PD (Vilariño‐Güell et al. 2011) and α‐Syn degradation is retromer‐dependent (Miura et al. 2014). One major cargo of retromer‐dependent trafficking is the CI‐MPR. Impairment of CI‐MPR transport affects the localization of acidic hydrolases such as cathepsin D to the lysosome (Miura et al. 2014). Interestingly, cathepsin D is the lysosomal hydrolase cleaving the bulk part of α‐Syn (Cullen et al. 2009; Sevlever et al. 2008). To investigate whether DNAJC13(N855S) expression affects the localization of retromer cargoes such as CI‐MPR, HEK293 A cells were transfected with GFP‐tagged DNAJC13 or DNAJC13(N855S). When comparing the localization of wild‐type DNAJC13 and DNAJC13(N855S), we did not observe an overt mislocalization of the mutant protein compared to DANJC13(WT) (Figure 3A; Figure S3). Both GFP‐tagged proteins co‐localize with SNX1 and VPS35, components of the retromer complex, as well as with WASH1, which is part of the WASH complex. Furthermore, both proteins are associated with the early endosome, but not with the late endosome as they co‐localize with EEA1 but not with RAB7, respectively (Figure S3). Our data confirm previous observations, that DNJC13(N855S) and the mutant localize at the recycling endosome (Fujibayashi et al. 2008; Girard et al. 2005; Yoshida et al. 2018). The CI‐MPR is one of the prominent retromer cargoes, which shuttles between the Golgi complex and endosomal compartments. Under steady‐state conditions and the expression of DNAJC13‐GFP, the majority of the CI‐MPR was accumulated in the perinuclear region, representing the Golgi apparatus. CI‐MPR and DNAJC13‐GFP partially colocalized (Figure 3A), indicating a partial localization of the CI‐MPR at endosomal membranes. In contrast, expression of DNAJC13(N855S) resulted in a more dispersed distribution of CI‐MPR concomitant with reduced co‐localization of DNAJC13(N855S) and CI‐MPR (Figure 3A). This is in line with recent data presented by Yoshida and co‐workers (Yoshida et al. 2024). Dispersed CI‐MPR distribution was previously recognized upon transient *DNAJC13/rme‐8* knockdown (Besemer et al. 2021; Girard et al. 2005; Popoff et al. 2009). A redistribution of CI‐MPR was also observed upon expression of the VPS35(D620N) mutant. In contrast to the expression of DNAJC13(N855S), CI‐MPR is more concentrated in the perinuclear space and seems to be trapped in the Golgi apparatus upon mutant VPS35(D620N) expression (Follett et al. 2014). To analyze Golgi‐association upon DNAJC13(N855S) expression, co‐localization of CI‐MPR and the trans‐Golgi network marker TGN46 was determined. Interestingly, co‐localization of CI‐MPR and TGN46 was comparable between DNAJC13(WT) and DNAJC13(N855S) expression (Figure 3B). These data indicate that DNAJC13(N855S) affects CI‐MPR localization differently than VPS35(D620N).

### 3.4 Altered CI‐MPR Distribution Does Not Lead to Altered Processing of Cathepsin D

α‐Syn is known to be degraded by autophagy as well as by lysosomes. In terms of lysosomal degradation, the bulk part of α‐Syn is cleaved by cathepsin D (Munsie et al. 2015; Sevlever et al. 2008), which is transported as a precursor by the CI‐MPR toward the lysosome (Tayebi et al. 2020). It is of note that cathepsin activity is inhibited in cell and animal models that show α‐Syn aggregation (Drobny et al. 2023). Since recycling of the CI‐MPR is mediated by the retromer complex and the trafficking of cathepsin D is CI‐MPR‐dependent, we analyzed the influence of DNAJC13 on cathepsin D localization and activity. The lysosomal enriched fractions from DNAJC13 stable knockdown and control cells were LAMP1 and LAMP2A positive, whereas tubulin and DNAJC13 were not identified by Western blot (Figure S4A), indicating proper separation of lysosomal fractions. Interestingly, protein levels of cathepsin D remained unchanged comparing total lysates (Figure S5) or lysosome‐enriched fractions (Figure 3C,D). As a measure for cathepsin D processing, the ratio of mature cathepsin D and pro‐cathepsin D was determined in lysosomal‐enriched fractions (Figure 3C,E). Based on this measure, the processing of cathepsin D was not altered in stable knockdown cells compared to controls. Furthermore, the re‐expression of DNAJC13(WT) or its mutant variant in cells with reduced DNAJC13 levels did not significantly alter cathepsin D levels when analyzing total lysates (Figure S5). To determine cathepsin D activity, the cleavage of a fluorogenic substrate specific for cathepsin D/E by lysosome‐enriched fractions was determined. The signal was detected over time, whereby the slope of the graph represents cathepsin D/E activity. Cathepsin D/E activity was not affected by reduced DNAJC13 levels in two independent clones (Figure 3F,G). Furthermore, in a second set of experiments employing a substrate specific for different cathepsins like cathepsin B, C, L, and K, the activity of these cathepsins was independent of DNAJC13 protein levels as well (Figure S4C,D). Both experiments were controlled by the specific inhibitors pepstatin A (cathepsin D and other aspartyl proteases) and E‐64‐D (cysteine proteases), respectively. It is of note that expression and protein levels of LAMP2A, an adapter for chaperone‐mediated autophagy, remained unchanged in DNAJC13 knockdown cells, even during re‐expression of wild‐type or mutant DNAJC13 (Figure S5).

### 3.5 Stable Knockdown of DNAJC13 Leads to Downregulation of Genes Associated With Vesicular Fusion‐ and Budding Dynamics

Different sets of evidence, including from our lab, suggest an involvement of DNAJC13 in retromer‐dependent trafficking and in modulating autophagy (Besemer et al. 2021; Swords et al. 2023). However, the distinct role of DNAJC13 in these processes remains at least in part elusive. Although several lines of evidence demonstrate whole genome transcriptional changes under conditions of impaired retromer function (Connor‐Robson et al. 2019; Daly et al. 2023; Y. Liu et al. 2021; Mishra et al. 2022), we intentionally performed an expression profiling of autophagy‐ and endosome‐related genes (Table S1) and compared wild‐type to stable DNAJC13 knockdown cells under basal conditions to gain further insight on the role of DNAJC13 in autophagy. While it is formally possible that some expression changes might be caused by off‐target effects of the *DNAJC13* shRNA, several controls, including HSP90 and p53, argue against a global transcriptional dysregulation in response to the stable expression of this shRNA.

**Figure 4:**
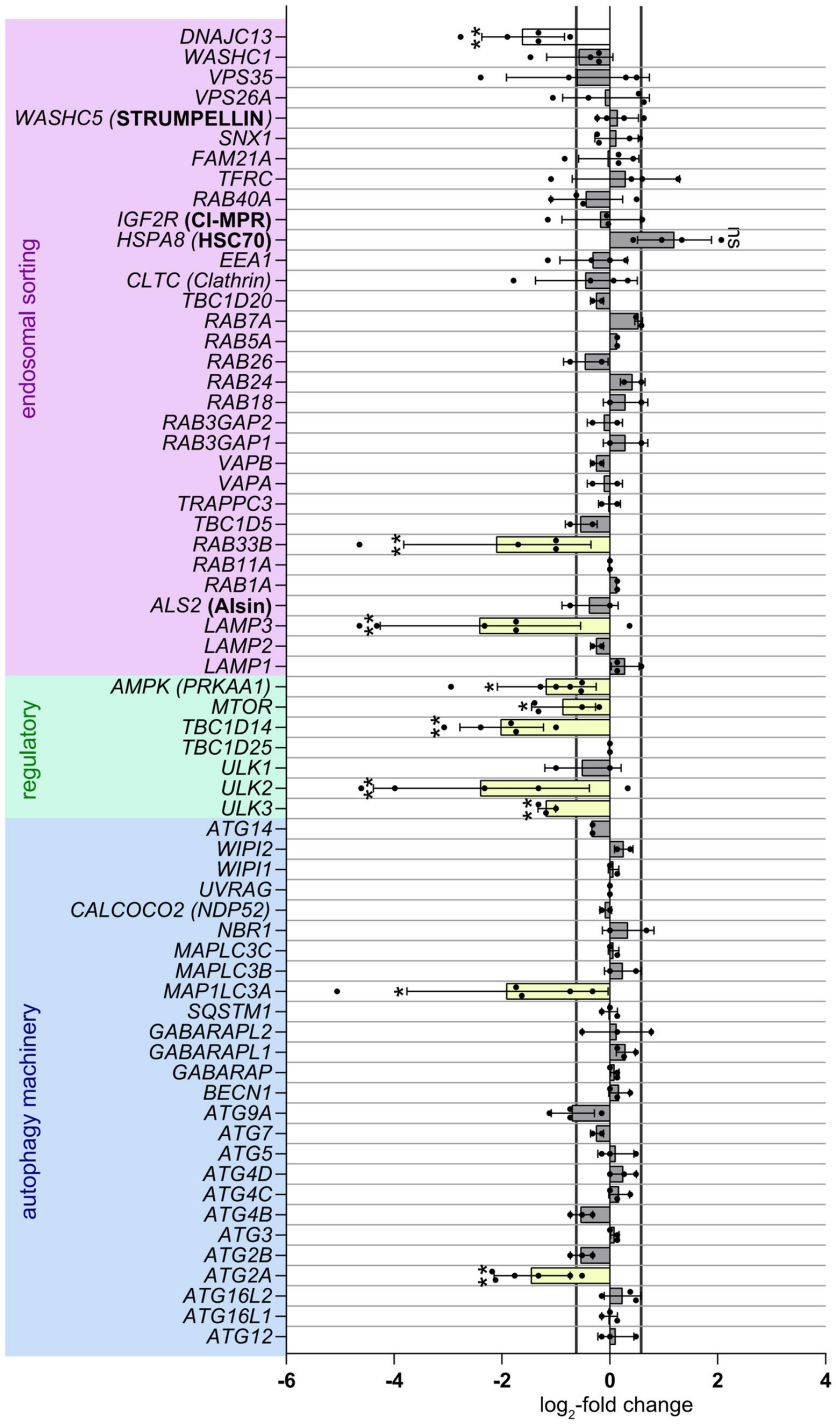
Several genes regulating vesicular fusion‐ and fission dynamics are repressed upon stable knockdown of DNAJC13. mRNA‐expression was determined using qRT‐PCR. RNA was isolated from regular HEK293T and stable *DNAJC13*‐knockdown cells. Subsequently, cDNA synthesis was performed with oligo‐dT‐primers, following qRT‐PCR. *DNA‐Methyl‐Transferase 1 (DNMT1)* served as housekeeping gene. RQ below 0.65 was considered as downregulation, values above 0.5 were considered upregulated. (mean ± SD; *n* = 4–8, *t*‐test: *p < 0.05; **p < 0.01; ***p < 0.001).

A defined set of genes appeared downregulated, but none of them, except *HSPA8* (HSC70), were upregulated (Figure 4). The increased expression of *HSPA8* (HSC70), although not significant, could suggest a compensatory mechanism considering DNAJC13 acting as a co‐chaperone of HSC70. Nonetheless, the list of downregulated genes included autophagy‐activating and autophagy‐inhibiting genes, suggesting the activation of compensatory mechanisms. On the one hand, we found *AMPK, ULK2*, and *ULK3* downregulated that, when activated, induce autophagic flux on different regulatory levels. On the other hand, the expression levels of *MTOR*, a central inhibitor of autophagy, and *TBC1D14*, an inhibitor of ATG9A vesicle traffic, were downregulated, suggesting an increase in autophagic flux via reduced inhibition.

Within the group of endosome‐related genes, the small GTPase *RAB33B* and *LAMP3* were significantly downregulated (Figure 4). Interestingly, RAB33B promotes autophagic flux by regulating the fusion of the autophagosome with the lysosome (Itoh et al. 2011), whereas LAMP3 inhibits autophagy (Tanaka et al. 2022). It is of note that the expression of genes related to the retromer and WASH complex as well as other genes directly interacting with DNAJC13 were not affected by DNAJC13 knockdown (Figure 4; Figure S6A,B). Finally, two genes of the autophagy core machinery instrumental in autophagosomal biogenesis, *ATG2* and *MAP1LC3A*, were downregulated. ATG2 interacts with ATG9A and regulates its retrograde recycling (Reggiori et al. 2004; Wang et al. 2001), whereas LC3A is mediating membrane integrity of autophagosomes (Javed et al. 2023). To exclude a clonal effect, the significantly regulated genes were verified in an independent DNAJC13 knockdown clone (Figure S6).

## 4 Discussion

It is a matter of debate whether DNAJC13(N855S) mediates toxicity by a loss of function or by the acquisition of a toxic quality. Our data support the view that loss of DNAJC13 function(s) is at least partially responsible for cellular toxicity. On the one hand, DNAJC13(N855S) is less stable than the wild‐type protein (Figure 1) resulting in reduced protein levels. On the other hand, mutant DNAJC13 is functionally impaired regarding its ability to positively modulate autophagy. This is supported by data that the re‐expression of the mutant protein cannot restore the autophagic flux phenotype in cells with chronically reduced DNAJC13 protein levels (Figure 2). Furthermore, the transient overexpression of DNAJC13(N855S) did not result in the formation of aggregates (Figure 1), suggesting that the mutant variant is not an intrinsically aggregation‐prone protein and might interfere with cellular homeostasis in this way. Nonetheless, DNAJC13 has been colocalized with a subset of Lewy bodies in patient tissues (Vilariño‐Güell et al. 2013) suggesting that the mutant protein might get trapped in aggregates under conditions of continued cellular stress in an aged tissue. Further investigations are necessary to analyze how the change of side chains from asparagine to serine modifies protein stability.

The observation that aggregated α‐Syn, even when applied extracellularly, inhibits its lysosomal degradation (Drobny et al. 2023; Hoffmann et al. 2019), and the identification of several PD‐associated genes related to the endo‐lysosomal system point toward a significant role of this pathway in the pathogenesis of the disease (Vidyadhara et al. 2019). One of these genes is *DNAJC13*, coding for a protein that interacts with the retromer as well as with the WASH complex. Retromer‐mediated dysfunction of endo‐lysosomal dynamics is an emerging pathomechanism of PD, resulting in the appearance of α‐Syn accumulations (Yoshida and Hasegawa 2022). One possible mechanism might be an inefficient transport of cathepsin D, which facilitates α‐Syn lysosomal degradation. Whereas α‐Syn itself enters the lysosome via LAMP2A and is degraded by chaperone‐mediated autophagy (Cui et al. 2018) cathepsin D is directed toward the lysosome in a retromer‐dependent manner via CI‐MPR (Arighi et al. 2004; Cui et al. 2018; Sevlever et al. 2008). In the case of the PD‐related VPS35(D620N) mutation (Vilariño‐Güell et al. 2011), a disrupted recycling of CI‐MPR was observed, which is supposedly due to perturbed interaction between retromer and WASH complex (McGough et al. 2014; Zavodszky et al. 2014).

We observed a peripheral dispersion of CI‐MPR upon the transient knockdown of DNAJC13 (Besemer et al. 2021) and the overexpression of DNAJC13(N855S) in normal HEK293 cells (Figure 3A,B), confirming recent data from Yoshida and co‐workers (Yoshida et al. 2024). It is of note that the persistent knockdown of DNAJC13 did not lead to altered lysosomal cathepsin D levels nor to a reduced activity of cathepsin D in lysosomal fractions (Figure 3C–G). This phenotype seems to differ from the one observed under VPS35(D620N) expression. In this case, the distribution of CI‐MPR is either unchanged (Kvainickas et al. 2017; Simonetti et al. 2017; Tsika et al. 2014) or getting trapped within the Golgi apparatus (Follett et al. 2014), followed by a disrupted transport of pro‐cathepsin D to the lysosome (Follett et al. 2014). However, peripheral dispersion of CI‐MPR similar to the herein observed effect was described upon knockdown of components of the WASH complex (Gomez and Billadeau 2009) and the knockdown of SNX1 (Kvainickas et al. 2017). Furthermore, SNX has a function in CI‐MPR trafficking which is independent of the core VPS‐trimer (Kvainickas et al. 2017; Simonetti et al. 2017). Bearing in mind that the knockdown of DNAJC13 as well as the expression of DNAJC13(N855S) resulted in increased SNX1‐positive endosomal tubulation (Follett et al. 2019; Freeman et al. 2014), it appears reasonable that DNAJC13 might participate in coordinating the WASH complex‐ and SNX1‐dependent export of CI‐MPR from the early toward the Golgi apparatus.

In line with this hypothesis, we identified several downregulated genes under stable knockdown conditions of DNAJC13, all of them playing a crucial role in vesicular fusion and budding dynamics or being directly involved in autophagosome formation (Figure 4). All of the regulated genes are involved in ATG9A trafficking as will be discussed below, and all, but one of these, namely TBC1D14, largely have a positive effect on autophagy. Interestingly, the regulated genes seemingly do not reside in the early endosome in the context of ATG9A trafficking. It is of note, that loss of retromer or retromer‐associated proteins like VPS35, SORL1, or LRRK2 result in cell type‐specific transcriptional changes (Connor‐Robson et al. 2019; Daly et al. 2023; Y. Liu et al. 2021; Mishra et al. 2022). Although it seems unlikely that retromer proteins act as transcription factors, a common theme mediating transcriptional changes is an impairment of cell surface receptors recycling with might, at least in part, explain the cell type specificity (Mishra et al. 2022; Rahman et al. 2020). In addition, a recycling‐independent function of retromer is linked to the regulation of the lysosome‐associated transcription factor TFEB. Here, retromer is involved in maintaining endosomal RAB7 domains. RAB7 governs cytosolic mTORC‐association with and phosphorylation of TFEB which translocates into the nucleus upon lysosomal stress or retromer dysfunction (Kvainickas et al. 2019).

LC3A is a member of the ATG8 protein family and, among other functions, interacts with the mitophagy‐receptor FUNDC (L. Liu et al. 2012) and contributes to the integrity and the sealing of autophagosomal membranes by interacting with the ESCRT‐I component VPS37A (Graef 2018). It is now tempting to hypothesize that DNAJC13 affects LC3A by interfering with the trafficking of VPS37A as DNAJC13 has been shown to balance ESCRT‐driven assembly of recycling and degradative sub‐endosomal domains (Norris et al. 2017). ATG2A was shown to promote vesicular fusion, supported by its rod‐shaped structure (Graef 2018). It localizes on the pre‐autophagic structure and lipid droplets (Velikkakath et al. 2012), and directly interacts with ATG9A (Wang et al. 2001). More specifically, ATG2 and ATG9A are located at the interface between the endoplasmic reticulum and the phagophore (Gómez‐Sánchez et al. 2018) where they form a heterotetrameric complex facilitating lipid flow toward the phagophore and the equilibration of its lipid bilayer (Van Vliet et al. 2022). In addition, ATG2 regulates the retrograde recycling of ATG9A (Wang et al. 2001). TBC1D14 which is believed to be a GAP (GTPase‐activating proteins) for the RAB family of small GTPases, is a key player in regulating ATG9A trafficking through the recycling endosome and in maintaining the so‐called ATG9 compartment (Lamb et al. 2016; Longatti et al. 2012). It negatively regulates ATG9A transport toward the phagophore and thus autophagic flux (Lamb et al. 2016). The reduced expression of TBC1D14 in cells with persistently reduced DNAJC13 levels might be a compensatory mechanism and a consequence of an altered endosomal subdomain architecture. Moreover, TBC1D14 overexpression displayed a disruption of Tf‐R traffic through the recycling endosome (Longatti et al. 2012) comparable to a knockdown of DNAJC13 (Girard et al. 2005). RAB33B is facilitating Golgi stack organization and is part of the traffic machinery from the Golgi apparatus toward the endoplasmic reticulum (S. Liu and Storrie 2012). RAB33B is additionally directly involved in autophagosome formation through physical interaction with ATG16L (Itoh et al. 2008) and recruitment of the ATG5‐12/16L‐complex to phagophores (Pantoom et al. 2021). LAMP3 is a lysosomal protein and considered a risk factor for PD (Pihlstrøm et al. 2013). The knockdown of LAMP3 as well as its overexpression disrupt autophagic degradation (Dominguez‐Bautista et al. 2015; Tanaka et al. 2022). The molecular basis for this is not fully understood. On the one hand, overexpression of LAMP3 seems to inhibit the fusion of autophagosomes and lysosomes which results in an accumulation of autophagosomes (Tanaka et al. 2022). On the other hand, the knockdown of LAMP3 inhibits the autophagy induction after proteasomal inhibition but not after general autophagy induction through amino acid starvation (Dominguez‐Bautista et al. 2015).

Analyzing the expression of several kinases involved in regulating autophagy, we observed that AMPK and MTOR levels were reduced in DNAJC13 knockdown cells. AMPK and MTOR are sensors for energy and nutrient supply, respectively, whereby their activities having opposing effects on autophagy. AMPK positively regulates autophagy by directly activating ULK1/ULK2 and by inhibiting MTOR (Alers et al. 2012). Interestingly, the ULK1‐dependent localization of ATG9A depends on the AMPK‐mediated phosphorylation of ULK1 (Mack et al. 2012). Active MTOR phosphorylates ULK1 and ULK2 at different sites than AMPK resulting in autophagy inhibition (Hosokawa et al. 2009). It is of note that ULK2 but not ULK1 showed reduced expression levels in DNAJC13 knockdown cells. ULK1 and ULK2 share a high homology in the catalytic and the C‐terminal domains. They both form a complex with FIP200 and ATG13 (Chan et al. 2009; Jung et al. 2009), locate on the omegasome (Chan et al. 2007; Cheong and Klionsky 2008) and regulate the phosphorylation of proteins containing PI(3)P‐effector domains, such as ATG2 (Papinski et al. 2014). Despite redundant functions within the autophagy pathway under some conditions (Lee and Tournier 2011; McAlpine et al. 2013), ULK1 and ULK2 display specific functions in certain cell types and adult tissues (Chan et al. 2007; Demeter et al. 2020; Harris et al. 2022). ULK2 knockdown in HEK293 cells did not alter general autophagic flux (Chan et al. 2007) but it might be involved in special types of selective autophagy in these cells (Demeter et al. 2020). ULK3 belongs to the ATG1 protein family of kinases like ULK1/ULK2. Besides promoting budding dynamics by phosphorylation of the ESCRT‐III‐complex (Caballe et al. 2015), it regulates autophagy independent of ULK1/2 in *drosophila melanogaster* (Braden and Neufeld 2016). ULK3 is upregulated upon serum starvation and its overexpression increases autophagic flux (Goruppi et al. 2017; Young et al. 2009). Interestingly, the starvation‐induced ULK3 activity inhibits LC3B expression by DNMT3A‐mediated methylation of the *LC3B* promotor (González‐Rodríguez et al. 2022).

Besides its role in autophagy induction, MTOR activity is involved in regulating the autophagic lysosome reformation (ALR) (Nanayakkara et al. 2023). The observed reduced levels of MTOR transcripts in DNAJC13 knockdown cells, might impair ALR and thus, as a feedback loop, autophagic flux. Recent evidence suggests that DNAJC13 is also directly involved in ALR (Swords et al. 2023). DNAJC13/RME‐8 knockdown in mammalian neuronal culture and in so‐called ALM neurons of *C. elegans* resulted in reduced autophagic flux concomitant with elongated autolysosomal tubules. The formation of these elongated tubules might be mediated by the role of DNAJC13/RME‐8 as a co‐chaperone for HSC70 controlling clathrin dynamics (Swords et al. 2023).

**Figure 5:**
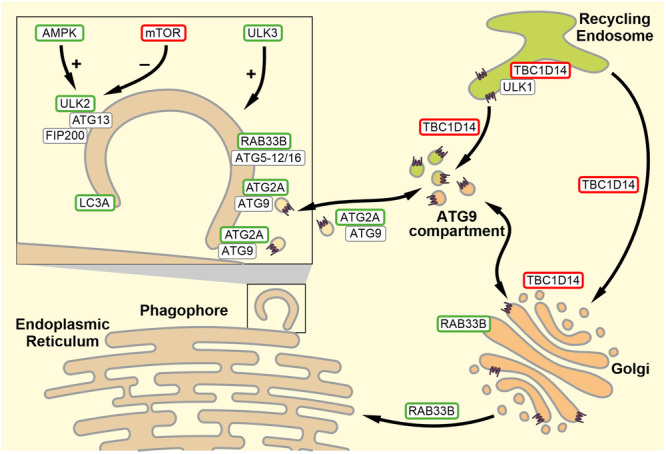
Functional interaction of regulated genes in stable DNAJC13 knockdown cells. The expression of a set of genes (boxed; see Figure 4) related to vesicle transport and autophagosome biogenesis was downregulated in cells with persistent reduced DNAJC13 protein levels compared to normal HEK293 cells. Interestingly, none of the analyzed genes were upregulated. Genes in green and red boxes positively and negatively affect autophagy, respectively. See text for details.

In summary, all genes that were downregulated upon chronically reduced DNAJC13 levels are closely related to the formation of autophagosomes and/or ATG9A trafficking (Figure 5). This is consistent with our previous observations that the transient knockdown of DNAJC13 affected ATG9A localization at the recycling endosome and resulted in a reduced co‐localization of ATG9A with LC3B (Besemer et al. 2021). Considering missing evidence concerning a physical interaction between DNAJC13 and ATG9A (data not shown), ATG9A mislocalization through reduced DNAJC13 levels might be caused by its interference with the conserved budding machinery. DNAJC13 is involved in clathrin dynamics and affects clathrin distribution, possibly by its co‐chaperone activity (Chang et al. 2004; Girard et al. 2005). Having said this, the lack of DNAJC13/RME‐8 resulted in an accumulation of clathrin in the endosomal compartment, with in turn jeopardizes ALR (Swords et al. 2023), highlighting the role of DNAJC13/RME‐8 in recycling processes in the early endosomal compartment and thereby affecting autophagy. Moreover, as DNAJC13 is central in forming endosomal microdomains (Norris et al. 2017; Norris et al. 2022), reduced DNAJC13 levels might affect domain organization and protein sorting of the recycling endosome. This might directly interfere with ATG9A trafficking or affect the distribution of one or several components regulating ATG9A function and transport. It is of note that the DNAJC13(N855S) variant is not able to compensate for these effects upon transient expression and might thus impair one route of α‐Syn degradation.

## Author Contributions

Conceptualization and design: Anna Stein, Christian Behl, and Albrecht M. Clement. Acquisition of data, analysis, and interpretation: Anna Stein, Stella Vo, Christian Freese, Joram Kluge, Joanna Maus, Ingrid Koziollek‐Drechsler, Beate Silva, and Albrecht M. Clement. Drafting and writing the manuscript: Anna Stein and Albrecht M. Clement. Critical reading and important intellectual input: (Joram Kluge, Joanna Maus, Christian Freese, and Christian Behl). Approval of the final version (all authors).

## Conflicts of Interest

The authors declare no conflicts of interest.

## Supporting information


**Supplemental Figure 1:** HSP90‐levels are stable upon inhibition of translation independent of DNAJC13 variant expression. **Supplemental Figure 2:** DNAJC13 itself is not a client of autophagic degradation. **Supplemental Figure 3:** The mutation in DNAJC13(N855S) does not result in an overt altered subcellular localization. **Supplemental Figure 4:** Cathepsin D levels or cathepsin D activity in lysosomal enriched fraction are not affected by knockdown of DNAJC13. **Supplemental Figure 5:** Cathepsin D, LAMP2A‐level and the processing of pro‐cathepsin D remain unaffected under stable knockdown (KD) of DNAJC13 and re‐expression of wildtype DNAJC13 or DNAJC13(N855S). **Supplemental Figure 6:** The downregulation of the identified genes upon stable knockdown of DNAJC13 can be validated in other independent DNAJC13 knockdown cell lines. The mRNA‐expression of DNAJC13 associated genes remains largely unchanged. **Table 1:** Primer for RQ‐PCR.

## Data Availability

The data that support the findings of this study are available from the corresponding author (AMC) upon reasonable request.

## References

[jcp70074-bib-0001] Alers, S. , A. S. Löffler , S. Wesselborg , and B. Stork . 2012. “Role of AMPK‐mTOR‐Ulk1/2 in the Regulation of Autophagy: Cross Talk, Shortcuts, and Feedbacks.” Molecular and Cellular Biology 32, no. 1: 2–11. 10.1128/MCB.06159-11. 22025673 PMC3255710

[jcp70074-bib-0002] Arighi, C. N. , L. M. Hartnell , R. C. Aguilar , C. R. Haft , and J. S. Bonifacino . 2004. “Role of the Mammalian Retromer in Sorting of the Cation‐Independent Mannose 6‐Phosphate Receptor.” Journal of Cell Biology 165, no. 1: 123–133. 10.1083/jcb.200312055. 15078903 PMC2172094

[jcp70074-bib-0003] Besemer, A. S. , J. Maus , M. Ax , et al. 2021. “Receptor‐Mediated Endocytosis 8 (RME‐8)/DNAJC13 Is a Novel Positive Modulator of Autophagy and Stabilizes Cellular Protein Homeostasis.” Cellular and Molecular Life Sciences: CMLS 78, no. 2: 645–660. 10.1007/s00018-020-03521-y. 32322926 PMC7873018

[jcp70074-bib-0004] Blauwendraat, C. , M. A. Nalls , and A. B. Singleton . 2020. “The Genetic Architecture of Parkinson′s Disease.” Lancet Neurology 19, no. 2: 170–178. 10.1016/S1474-4422(19)30287-X. 31521533 PMC8972299

[jcp70074-bib-0005] Braden, C. R. , and T. P. Neufeld . 2016. “Atg1‐Independent Induction of Autophagy by the Drosophila Ulk3 Homolog, ADUK.” FEBS Journal 283, no. 21: 3889–3897. 10.1111/febs.13906. 27717182 PMC5123689

[jcp70074-bib-0006] Caballe, A. , D. M. Wenzel , M. Agromayor , et al. 2015. “Ulk3 Regulates Cytokinetic Abscission by Phosphorylating ESCRT‐III Proteins.” eLife 4: e06547. 10.7554/eLife.06547. 26011858 PMC4475061

[jcp70074-bib-0007] Chan, E. Y. , S. Kir , and S. A. Tooze . 2007. “Sirna Screening of the Kinome Identifies ULK1 as a Multidomain Modulator of Autophagy.” Journal of Biological Chemistry 282, no. 35: 25464–25474. 10.1074/jbc.M703663200. 17595159

[jcp70074-bib-0008] Chan, E. Y. , A. Longatti , N. C. McKnight , and S. A. Tooze . 2009. “Kinase‐Inactivated ULK Proteins Inhibit Autophagy via Their Conserved C‐Terminal Domains Using an Atg13‐Independent Mechanism.” Molecular and Cellular Biology 29, no. 1: 157–171. 10.1128/MCB.01082-08. 18936157 PMC2612494

[jcp70074-bib-0009] Chang, H. C. , M. Hull , and I. Mellman . 2004. “The J‐Domain Protein Rme‐8 Interacts With Hsc70 to Control Clathrin‐Dependent Endocytosis in *Drosophila* .” Journal of Cell Biology 164, no. 7: 1055–1064. 10.1083/jcb.200311084. 15051737 PMC2172058

[jcp70074-bib-0010] Cheong, H. , and D. J. Klionsky . 2008. “Dual Role of Atg1 in Regulation of Autophagy‐Specific PAS Assembly in *Saccharomyces cerevisiae* .” Autophagy 4, no. 5: 724–726. 10.4161/auto.6375. 18552550

[jcp70074-bib-0011] Connor‐Robson, N. , H. Booth , J. G. Martin , et al. 2019. “An Integrated Transcriptomics and Proteomics Analysis Reveals Functional Endocytic Dysregulation Caused by Mutations in LRRK2.” Neurobiology of Disease 127: 512–526. 10.1016/j.nbd.2019.04.005. 30954703 PMC6597903

[jcp70074-bib-0012] Craig, E. A. , and J. Marszalek . 2017. “How Do J‐Proteins Get Hsp70 to Do So Many Different Things?” Trends in Biochemical Sciences 42, no. 5: 355–368. 10.1016/j.tibs.2017.02.007. 28314505 PMC5409888

[jcp70074-bib-0013] Cui, Y. , Z. Yang , and R. D. Teasdale . 2018. “The Functional Roles of Retromer in Parkinson′s Disease.” FEBS Letters 592, no. 7: 1096–1112. 10.1002/1873-3468.12931. 29210454

[jcp70074-bib-0014] Cullen, V. , M. Lindfors , J. Ng , et al. 2009. “Cathepsin D Expression Level Affects Alpha‐Synuclein Processing, Aggregation, and Toxicity In Vivo.” Molecular Brain 2: 5. 10.1186/1756-6606-2-5. 19203374 PMC2644690

[jcp70074-bib-0015] Daly, J. L. , C. M. Danson , P. A. Lewis , et al. 2023. “Multi‐Omic Approach Characterises the Neuroprotective Role of Retromer in Regulating Lysosomal Health.” Nature Communications 14, no. 1: 3086. 10.1038/s41467-023-38719-8. PMC10227043 37248224

[jcp70074-bib-0016] Demeter, A. , M. C. Romero‐Mulero , L. Csabai , et al. 2020. “Ulk1 and ULK2 Are Less Redundant Than Previously Thought: Computational Analysis Uncovers Distinct Regulation and Functions of These Autophagy Induction Proteins.” Scientific Reports 10, no. 1: 10940. 10.1038/s41598-020-67780-2. 32616830 PMC7331686

[jcp70074-bib-0017] Deng, H. X. , Y. Shi , Y. Yang , et al. 2016. “Identification of TMEM230 Mutations in Familial Parkinson′s Disease.” Nature Genetics 48, no. 7: 733–739. 10.1038/ng.3589. 27270108 PMC6047531

[jcp70074-bib-0018] Dominguez‐Bautista, J. A. , M. Klinkenberg , N. Brehm , et al. 2015. “Loss of Lysosome‐Associated Membrane Protein 3 (LAMP3) Enhances Cellular Vulnerability Against Proteasomal Inhibition.” European Journal of Cell Biology 94, no. 3–4: 148–161. 10.1016/j.ejcb.2015.01.003. 25681212

[jcp70074-bib-0019] Drobny, A. , F. A. Boros , D. Balta , et al. 2023. “Reciprocal Effects of Alpha‐Synuclein Aggregation and Lysosomal Homeostasis in Synucleinopathy Models.” Translational Neurodegeneration 12, no. 1: 31. 10.1186/s40035-023-00363-z. 37312133 PMC10262594

[jcp70074-bib-0020] Follett, J. , J. D. Fox , E. K. Gustavsson , et al. 2019. “DNAJC13 p.Asn855Ser, Implicated in Familial Parkinsonism, Alters Membrane Dynamics of Sorting Nexin 1.” Neuroscience Letters 706: 114–122. 10.1016/j.neulet.2019.04.043. 31082451

[jcp70074-bib-0021] Follett, J. , S. J. Norwood , N. A. Hamilton , et al. 2014. “The Vps35 D620N Mutation Linked to Parkinson′s Disease Disrupts the Cargo Sorting Function of Retromer.” Traffic (Copenhagen, Denmark) 15, no. 2: 230–244. 10.1111/tra.12136. 24152121

[jcp70074-bib-0022] Freeman, C. L. , G. Hesketh , and M. N. J. Seaman . 2014. “RME‐8 Coordinates the WASH Complex With the Retromer SNX‐BAR Dimer to Control Endosomal Tubulation.” Journal of Cell Science 1: 2053–2070. 10.1242/jcs.144659. PMC4004978 24643499

[jcp70074-bib-0023] Fujibayashi, A. , T. Taguchi , R. Misaki , et al. 2008. “Human RME‐8 Is Involved in Membrane Trafficking Through Early Endosomes.” Cell Structure and Function 33, no. 1: 35–50. 10.1247/csf.07045. 18256511

[jcp70074-bib-0024] Gagliardi, M. , G. Annesi , R. Procopio , et al. 2018. “Dnajc13 Mutation Screening in Patients With Parkinson′s Disease From South Italy.” Parkinsonism & Related Disorders 55: 134–137. 10.1016/j.parkreldis.2018.06.004. 29887357

[jcp70074-bib-0025] Gialluisi, A. , M. G. Reccia , N. Modugno , et al. 2021. “Identification of Sixteen Novel Candidate Genes for Late Onset Parkinson′s Disease.” Molecular Neurodegeneration 16, no. 1: 35. 10.1186/s13024-021-00455-2. 34148545 PMC8215754

[jcp70074-bib-0026] Girard, M. , and P. S. McPherson . 2008. “RME‐8 Regulates Trafficking of the Epidermal Growth Factor Receptor.” FEBS Letters 582, no. 6: 961–966. 10.1016/j.febslet.2008.02.042. 18307993

[jcp70074-bib-0027] Girard, M. , V. Poupon , F. Blondeau , and P. S. McPherson . 2005. “The DnaJ‐Domain Protein RME‐8 Functions in Endosomal Trafficking.” Journal of Biological Chemistry 280, no. 48: 40135–40143. 10.1074/jbc.M505036200. 16179350

[jcp70074-bib-0028] Gomez, T. S. , and D. D. Billadeau . 2009. “A FAM21‐Containing WASH Complex Regulates Retromer‐Dependent Sorting.” Developmental Cell 17, no. 5: 699–711. 10.1016/j.devcel.2009.09.009. 19922874 PMC2803077

[jcp70074-bib-0029] Gomez‐Lamarca, M. J. , L. A. Snowdon , E. Seib , T. Klein , and S. J. Bray . 2015. “Rme‐8 Depletion Perturbs Notch Recycling and Predisposes to Pathogenic Signaling.” Journal of Cell Biology 210, no. 2: 303–318. 10.1083/jcb.201411001. 26169355 PMC4508892

[jcp70074-bib-0030] Gómez‐Sánchez, R. , J. Rose , R. Guimarães , et al. 2018. “Atg9 Establishes Atg2‐Dependent Contact Sites Between the Endoplasmic Reticulum and Phagophores.” Journal of Cell Biology 217, no. 8: 2743–2763. 10.1083/jcb.201710116. 29848619 PMC6080931

[jcp70074-bib-0031] González‐Rodríguez, P. , M. Cheray , L. Keane , P. Engskog‐Vlachos , and B. Joseph . 2022. “Ulk3‐Dependent Activation of GLI1 Promotes DNMT3A Expression Upon Autophagy Induction.” Autophagy 18, no. 12: 2769–2780. 10.1080/15548627.2022.2039993. 35226587 PMC9673947

[jcp70074-bib-0032] Goruppi, S. , M. G. Procopio , S. Jo , A. Clocchiatti , V. Neel , and G. P. Dotto . 2017. “The ULK3 Kinase Is Critical for Convergent Control of Cancer‐Associated Fibroblast Activation by CSL and GLI.” Cell Reports 20, no. 10: 2468–2479. 10.1016/j.celrep.2017.08.048. 28877478 PMC5616185

[jcp70074-bib-0033] Graef, M. 2018. “Membrane Tethering by the Autophagy ATG2A‐WIPI4 Complex.” Proceedings of the National Academy of Sciences of the United States of America 115, no. 42: 10540–10541. 10.1073/pnas.1814759115. 30275332 PMC6196487

[jcp70074-bib-0034] Gumpper, K. , M. Sermersheim , M. X. Zhu , and P. H. Lin . 2019. “Skeletal Muscle Lysosomal Function via Cathepsin Activity Measurement.” Methods in Molecular Biology (Clifton, N.J.) 1854: 35–43. 10.1007/7651_2017_64. PMC5828979 28842895

[jcp70074-bib-0035] Harris, M. P. , Q. J. Zhang , C. T. Cochran , et al. 2022. “Perinatal Versus Adult Loss of ULK1 and ULK2 Distinctly Influences Cardiac Autophagy and Function.” Autophagy 18, no. 9: 2161–2177. 10.1080/15548627.2021.2022289. 35104184 PMC9466614

[jcp70074-bib-0036] Harrison, M. S. , C. S. Hung , T. T. Liu , R. Christiano , T. C. Walther , and C. G. Burd . 2014. “A Mechanism for Retromer Endosomal Coat Complex Assembly With Cargo.” Proceedings of the National Academy of Sciences of the United States of America 111, no. 1: 267–272. 10.1073/pnas.1316482111. 24344282 PMC3890810

[jcp70074-bib-0037] Hoffmann, A. C. , G. Minakaki , S. Menges , et al. 2019. “Extracellular Aggregated Alpha Synuclein Primarily Triggers Lysosomal Dysfunction in Neural Cells Prevented by Trehalose.” Scientific Reports 9, no. 1: 544. 10.1038/s41598-018-35811-8. 30679445 PMC6345801

[jcp70074-bib-0038] Hosokawa, N. , T. Hara , T. Kaizuka , et al. 2009. “Nutrient‐Dependent mTORC1 Association With the ULK1‐Atg13‐FIP200 Complex Required for Autophagy.” Molecular Biology of the Cell 20, no. 7: 1981–1991. 10.1091/mbc.e08-12-1248. 19211835 PMC2663915

[jcp70074-bib-0039] Itoh, T. , N. Fujita , E. Kanno , A. Yamamoto , T. Yoshimori , and M. Fukuda . 2008. “Golgi‐Resident Small GTPase Rab33B Interacts With Atg16L and Modulates Autophagosome Formation.” Molecular Biology of the Cell 19, no. 7: 2916–2925. 10.1091/mbc.e07-12-1231. 18448665 PMC2441679

[jcp70074-bib-0040] Itoh, T. , E. Kanno , T. Uemura , S. Waguri , and M. Fukuda . 2011. “Oatl1, a Novel Autophagosome‐Resident Rab33B‐GAP, Regulates Autophagosomal Maturation.” Journal of Cell Biology 192, no. 5: 839–853. 10.1083/jcb.201008107. 21383079 PMC3051816

[jcp70074-bib-0041] Javed, R. , A. Jain , T. Duque , et al. 2023. “Mammalian ATG8 Proteins Maintain Autophagosomal Membrane Integrity Through ESCRTs.” EMBO Journal 42, no. 14: e112845. 10.15252/embj.2022112845. 37272163 PMC10350836

[jcp70074-bib-0042] Jung, C. H. , C. B. Jun , S. H. Ro , et al. 2009. “Ulk‐Atg13‐FIP200 Complexes Mediate mTOR Signaling to the Autophagy Machinery.” Molecular Biology of the Cell 20, no. 7: 1992–2003. 10.1091/mbc.e08-12-1249. 19225151 PMC2663920

[jcp70074-bib-0043] Kovtun, O. , N. Leneva , Y. S. Bykov , et al. 2018. “Structure of the Membrane‐Assembled Retromer Coat Determined by Cryo‐Electron Tomography.” Nature 561, no. 7724: 561–564. 10.1038/s41586-018-0526-z. 30224749 PMC6173284

[jcp70074-bib-0044] Kvainickas, A. , A. Jimenez‐Orgaz , H. Nägele , Z. Hu , J. Dengjel , and F. Steinberg . 2017. “Cargo‐Selective SNX‐BAR Proteins Mediate Retromer Trimer Independent Retrograde Transport.” Journal of Cell Biology 216, no. 11: 3677–3693. 10.1083/jcb.201702137. 28935632 PMC5674888

[jcp70074-bib-0045] Kvainickas, A. , H. Nägele , W. Qi , et al. 2019. “Retromer and TBC1D5 Maintain Late Endosomal RAB7 Domains to Enable Amino Acid‐Induced mTORC1 Signaling.” Journal of Cell Biology 218, no. 9: 3019–3038. 10.1083/jcb.201812110. 31431476 PMC6719456

[jcp70074-bib-0046] Lamb, C. A. , S. Nühlen , D. Judith , et al. 2016. “Tbc1d14 Regulates Autophagy via the TRAPP Complex and ATG9 Traffic.” EMBO Journal 35, no. 3: 281–301. 10.15252/embj.201592695. 26711178 PMC4741301

[jcp70074-bib-0047] de Lau, L. M. , and M. M. Breteler . 2006. “Epidemiology of Parkinson′s Disease.” Lancet Neurology 5, no. 6: 525–535. 10.1016/S1474-4422(06)70471-9. 16713924

[jcp70074-bib-0048] Lee, E. ‑J. , and C. Tournier . 2011. “The Requirement of Uncoordinated 51‐Like Kinase 1 (ULK1) and ULK2 in the Regulation of Autophagy.” Autophagy 7, no. 7: 689–695. 10.4161/auto.7.7.15450. 21460635 PMC3149696

[jcp70074-bib-0049] Li, C. , R. Ou , Y. Chen , et al. 2020. “Mutation Analysis of DNAJC Family for Early‐Onset Parkinson′s Disease in a Chinese Cohort.” Movement Disorders: Official Journal of the Movement Disorder Society 35, no. 11: 2068–2076. 10.1002/mds.28203. 32662538

[jcp70074-bib-0050] Liebl, M. P. , A. M. Kaya , S. Tenzer , et al. 2014. “Dimerization of Visinin‐Like Protein 1 Is Regulated by Oxidative Stress and Calcium and Is a Pathological Hallmark of Amyotrophic Lateral Sclerosis.” Free Radical Biology & Medicine 72: 41–54. 10.1016/j.freeradbiomed.2014.04.008. 24742816

[jcp70074-bib-0051] Lin, C. H. , P. L. Chen , C. H. Tai , et al. 2019. “A Clinical and Genetic Study of Early‐Onset and Familial Parkinsonism in Taiwan: An Integrated Approach Combining Gene Dosage Analysis and Next‐Generation Sequencing.” Movement Disorders: Official Journal of the Movement Disorder Society 34, no. 4: 506–515. 10.1002/mds.27633. 30788857 PMC6594087

[jcp70074-bib-0052] Liu, L. , D. Feng , G. Chen , Chen , et al. 2012. “Mitochondrial Outer‐Membrane Protein FUNDC1 Mediates Hypoxia‐Induced Mitophagy in Mammalian Cells.” Nature Cell Biology 14, no. 2: 177–185. 10.1038/ncb2422. 22267086

[jcp70074-bib-0053] Liu, S. , and B. Storrie . 2012. “Are Rab Proteins the Link Between Golgi Organization and Membrane Trafficking?” Cellular and Molecular Life Sciences: CMLS 69, no. 24: 4093–4106. 10.1007/s00018-012-1021-6. 22581368 PMC4080914

[jcp70074-bib-0054] Liu, Y. , H. Deng , L. Liang , et al. 2021. “Depletion of VPS35 Attenuates Metastasis of Hepatocellular Carcinoma by Restraining the Wnt/PCP Signaling Pathway.” Genes & Diseases 8, no. 2: 232–240. 10.1016/j.gendis.2020.07.009. 33997170 PMC8099696

[jcp70074-bib-0055] Longatti, A. , C. A. Lamb , M. Razi , S. Yoshimura , F. A. Barr , and S. A. Tooze . 2012. “TBC1D14 Regulates Autophagosome Formation via Rab11‐ and ULK1‐positive Recycling Endosomes.” Journal of Cell Biology 197, no. 5: 659–675. 10.1083/jcb.201111079. 22613832 PMC3365497

[jcp70074-bib-0056] Lucas, M. , D. C. Gershlick , A. Vidaurrazaga , A. L. Rojas , J. S. Bonifacino , and A. Hierro . 2016. “Structural Mechanism for Cargo Recognition by the Retromer Complex.” Cell 167, no. 6: 1623–1635. 10.1016/j.cell.2016.10.056. 27889239 PMC5147500

[jcp70074-bib-0057] Mack, H. I. , B. Zheng , J. M. Asara , and S. M. Thomas . 2012. “Ampk‐Dependent Phosphorylation of ULK1 Regulates ATG9 Localization.” Autophagy 8, no. 8: 1197–1214. 10.4161/auto.20586. 22932492 PMC3679237

[jcp70074-bib-0058] McAlpine, F. , L. E. Williamson , S. A. Tooze , and E. Y. Chan . 2013. “Regulation of Nutrient‐Sensitive Autophagy by Uncoordinated 51‐Like Kinases 1 and 2.” Autophagy 9, no. 3: 361–373. 10.4161/auto.23066. 23291478 PMC3590256

[jcp70074-bib-0059] McGough, I. J. , F. Steinberg , J. D. Barbuti , et al. 2014. “Retromer Binding to FAM21 and the WASH Complex Is Perturbed by the Parkinson Disease‐Linked VPS35(D620N) Mutation.” Current Biology: CB 24, no. 14: 1670–1676. 10.1016/j.cub.2014.06.024. 24980502 PMC4110399

[jcp70074-bib-0060] Mishra, S. , A. Knupp , M. P. Szabo , et al. 2022. “The Alzheimer′s Gene SORL1 Is a Regulator of Endosomal Traffic and Recycling in Human Neurons.” Cellular and Molecular Life Sciences: CMLS 79, no. 3: 162. 10.1007/s00018-022-04182-9. 35226190 PMC8885486

[jcp70074-bib-0061] Miura, E. , T. Hasegawa , M. Konno , et al. 2014. “Vps35 Dysfunction Impairs Lysosomal Degradation of α‐Synuclein and Exacerbates Neurotoxicity in a Drosophila Model of Parkinson′s Disease.” Neurobiology of Disease 71: 1–13. 10.1016/j.nbd.2014.07.014. 25107340

[jcp70074-bib-0062] Munsie, L. N. , A. J. Milnerwood , P. Seibler , et al. 2015. “Retromer‐Dependent Neurotransmitter Receptor Trafficking to Synapses Is Altered by the Parkinson′s Disease VPS35 Mutation p.D620N.” Human Molecular Genetics 24, no. 6: 1691–1703. 10.1093/hmg/ddu582. 25416282

[jcp70074-bib-0063] Nanayakkara, R. , R. Gurung , S. J. Rodgers , et al. 2023. “Autophagic Lysosome Reformation in Health and Disease.” Autophagy 19, no. 5: 1378–1395. 10.1080/15548627.2022.2128019. 36409033 PMC10240999

[jcp70074-bib-0064] Norris, A. , C. T. McManus , S. Wang , R. Ying , and B. D. Grant . 2022. “Mutagenesis and Structural Modeling Implicate RME‐8 IWN Domains as Conformational Control Points.” PLoS Genetics 18, no. 10: e1010296. 10.1371/journal.pgen.1010296. 36279308 PMC9642905

[jcp70074-bib-0065] Norris, A. , P. Tammineni , S. Wang , et al. 2017. “SNX‐1 and RME‐8 Oppose the Assembly of HGRS‐1/ESCRT‐0 Degradative Microdomains on Endosomes.” Proceedings of the National Academy of Sciences of the United States of America 114, no. 3: 307. 10.1073/pnas.1612730114. PMC5255583 28053230

[jcp70074-bib-0066] Pantoom, S. , G. Konstantinidis , S. Voss , et al. 2021. “Rab33b Recruits the ATG16L1 Complex to the Phagophore via a Noncanonical RAB Binding Protein.” Autophagy 17, no. 9: 2290–2304. 10.1080/15548627.2020.1822629. 32960676 PMC8496732

[jcp70074-bib-0067] Papinski, D. , M. Schuschnig , W. Reiter , et al. 2014. “Early Steps in Autophagy Depend on Direct Phosphorylation of Atg9 by the Atg1 Kinase.” Molecular Cell 53, no. 3: 471–483. 10.1016/j.molcel.2013.12.011. 24440502 PMC3978657

[jcp70074-bib-0068] Pfaffl, M. W. , G. W. Horgan , and L. Dempfle . 2002. “Relative Expression Software Tool (REST) for Group‐Wise Comparison and Statistical Analysis of Relative Expression Results in Real‐Time PCR.” Nucleic Acids Research 30, no. 9: e36. 10.1093/nar/30.9.e36. 11972351 PMC113859

[jcp70074-bib-0069] Pihlstrøm, L. , G. Axelsson , K. A. Bjørnarå , et al. 2013. “Supportive Evidence for 11 Loci From Genome‐Wide Association Studies in Parkinson′s Disease.” Neurobiology of Aging 34, no. 6: 1708. 10.1016/j.neurobiolaging.2012.10.019. 23153929

[jcp70074-bib-0070] Popoff, V. , G. A. Mardones , S. K. Bai , et al. 2009. “Analysis of Articulation Between Clathrin and Retromer in Retrograde Sorting on Early Endosomes.” Traffic (Copenhagen, Denmark) 10, no. 12: 1868–1880. 10.1111/j.1600-0854.2009.00993.x. 19874558

[jcp70074-bib-0071] Rahman, A. A. , A. Soto‐Avellaneda , H. Yong Jin , et al. 2020. “Enhanced Hyaluronan Signaling and Autophagy Dysfunction by VPS35 D620N.” Neuroscience 441: 33–45. 10.1016/j.neuroscience.2020.06.009. 32540366 PMC7390708

[jcp70074-bib-0072] Reggiori, F. , K. A. Tucker , P. E. Stromhaug , and D. J. Klionsky . 2004. “The Atg1‐Atg13 Complex Regulates Atg9 and Atg23 Retrieval Transport From the Pre‐Autophagosomal Structure.” Developmental Cell 6, no. 1: 79–90. 10.1016/s1534-5807(03)00402-7. 14723849

[jcp70074-bib-0073] Seaman, M. 2021. “The Retromer Complex: From Genesis to Revelations.” Trends in Biochemical Sciences 46, no. 7: 608–620. 10.1016/j.tibs.2020.12.009. 33526371

[jcp70074-bib-0074] Sevlever, D. , P. Jiang , and S. H. Yen . 2008. “Cathepsin D Is the Main Lysosomal Enzyme Involved in the Degradation of Alpha‐Synuclein and Generation of Its Carboxy‐Terminally Truncated Species.” Biochemistry 47, no. 36: 9678–9687. 10.1021/bi800699v. 18702517 PMC2630205

[jcp70074-bib-0075] Shi, A. , L. Sun , R. Banerjee , M. Tobin , Y. Zhang , and B. D. Grant . 2009. “Regulation of Endosomal Clathrin and Retromer‐Mediated Endosome to Golgi Retrograde Transport by the J‐Domain Protein RME‐8.” EMBO Journal 28, no. 21: 3290–3302. 10.1038/emboj.2009.272. 19763082 PMC2776105

[jcp70074-bib-0076] Simonetti, B. , C. M. Danson , K. J. Heesom , and P. J. Cullen . 2017. “Sequence‐Dependent Cargo Recognition by SNX‐BARs Mediates Retromer‐Independent Transport of CI‐MPR.” Journal of Cell Biology 216, no. 11: 3695–3712. 10.1083/jcb.201703015. 28935633 PMC5674890

[jcp70074-bib-0077] Swords, S. B. , N. Jia , A. Norris , J. Modi , Q. Cai , and B. D. Grant . 2024. “A Conserved Requirement for RME‐8/DNAJC13 in Neuronal Autophagic Lysosome Reformation.” Autophagy 20: 792–808. 10.1080/15548627.2023.2269028. 37942902 PMC11062384

[jcp70074-bib-0078] Tanaka, T. , B. M. Warner , D. G. Michael , et al. 2022. “Lamp3 Inhibits Autophagy and Contributes to Cell Death by Lysosomal Membrane Permeabilization.” Autophagy 18, no. 7: 1629–1647. 10.1080/15548627.2021.1995150. 34802379 PMC9298453

[jcp70074-bib-0079] Tayebi, N. , G. Lopez , J. Do , and E. Sidransky . 2020. “Pro‐Cathepsin D, Prosaposin, and Progranulin: Lysosomal Networks in Parkinsonism.” Trends in Molecular Medicine 26, no. 10: 913–923. 10.1016/j.molmed.2020.07.004. 32948448 PMC9067398

[jcp70074-bib-0080] Trinh, J. , K. Lohmann , H. Baumann , et al. 2019. “Utility and Implications of Exome Sequencing in Early‐Onset Parkinson′s Disease.” Movement Disorders: Official Journal of the Movement Disorder Society 34, no. 1: 133–137. 10.1002/mds.27559. 30537300 PMC8950081

[jcp70074-bib-0081] Tsika, E. , L. Glauser , R. Moser , et al. 2014. “Parkinson′s Disease‐Linked Mutations in VPS35 Induce Dopaminergic Neurodegeneration.” Human Molecular Genetics 23, no. 17: 4621–4638. 10.1093/hmg/ddu178. 24740878 PMC4119414

[jcp70074-bib-0082] Velikkakath, A. K. , T. Nishimura , E. Oita , N. Ishihara , and N. Mizushima . 2012. “Mammalian Atg2 Proteins Are Essential for Autophagosome Formation and Important for Regulation of Size and Distribution of Lipid Droplets.” Molecular Biology of the Cell 23, no. 5: 896–909. 10.1091/mbc.E11-09-0785. 22219374 PMC3290647

[jcp70074-bib-0083] Vidyadhara, D. J. , J. E. Lee , and S. S. Chandra . 2019. “Role of the Endolysosomal System in Parkinson′s Disease.” Journal of Neurochemistry 150, no. 5: 487–506. 10.1111/jnc.14820. 31287913 PMC6707858

[jcp70074-bib-0084] Vilariño‐Güell, C. , A. Rajput , A. J. Milnerwood , et al. 2014. “DNAJC13 Mutations in Parkinson Disease.” Human Molecular Genetics 23, no. 7: 1794–1801. 10.1093/hmg/ddt570. 24218364 PMC3999380

[jcp70074-bib-0085] Vilariño‐Güell, C. , C. Wider , O. A. Ross , et al. 2011. “VPS35 Mutations in Parkinson Disease.” American Journal of Human Genetics 89, no. 1: 162–167. 10.1016/j.ajhg.2011.06.001. 21763482 PMC3135796

[jcp70074-bib-0086] Van Vliet, A. R. , G. N. Chiduza , and S. L. Maslen , et al. 2022. “Atg9a and ATG2A Form a Heteromeric Complex Essential for Autophagosome Formation.” Molecular Cell 82, no. 22: 4324–4339. e8. 10.1016/j.molcel.2022.10.017. 36347259

[jcp70074-bib-0087] Wang, C. W. , J. Kim , W. P. Huang , et al. 2001. “Apg2 Is a Novel Protein Required for the Cytoplasm to Vacuole Targeting, Autophagy, and Pexophagy Pathways.” Journal of Biological Chemistry 276, no. 32: 30442–30451. 10.1074/jbc.M102342200. 11382760 PMC2737745

[jcp70074-bib-0088] Witan, H. , A. Kern , I. Koziollek‐Drechsler , R. Wade , C. Behl , and A. M. Clement . 2008. “Heterodimer Formation of Wild‐Type and Amyotrophic Lateral Sclerosis‐Causing Mutant Cu/Zn‐Superoxide Dismutase Induces Toxicity Independent of Protein Aggregation.” Human Molecular Genetics 17, no. 10: 1373–1385. 10.1093/hmg/ddn025. 18211954

[jcp70074-bib-0089] Xhabija, B. , and P. O. Vacratsis . 2015. “Receptor‐Mediated Endocytosis 8 Utilizes an N‐Terminal Phosphoinositide‐Binding Motif to Regulate Endosomal Clathrin Dynamics.” Journal of Biological Chemistry 290, no. 35: 21676–21689. 10.1074/jbc.M115.644757. 26134565 PMC4571890

[jcp70074-bib-0090] Yoshida, S. , and T. Hasegawa . 2022. “Beware of Misdelivery: Multifaceted Role of Retromer Transport in Neurodegenerative Diseases.” Frontiers in Aging Neuroscience 14: 897688. 10.3389/fnagi.2022.897688. 35601613 PMC9120357

[jcp70074-bib-0091] Yoshida, S. , T. Hasegawa , T. Nakamura , et al. 2024. “Dysregulation of SNX1‐Retromer Axis in Pharmacogenetic Models of Parkinson′S Disease.” Cell Death Discovery 10, no. 1: 290. 10.1038/s41420-024-02062-8. 38886344 PMC11183211

[jcp70074-bib-0092] Yoshida, S. , T. Hasegawa , M. Suzuki , et al. 2018. “Parkinson′S Disease‐Linked DNAJC13 Mutation Aggravates Alpha‐Synuclein‐Induced Neurotoxicity Through Perturbation of Endosomal Trafficking.” Human Molecular Genetics 27, no. 5: 823–836. 10.1093/hmg/ddy003. 29309590

[jcp70074-bib-0093] Young, A. R. , M. Narita , M. Ferreira , et al. 2009. “Autophagy Mediates the Mitotic Senescence Transition.” Genes & Development 23, no. 7: 798–803. 10.1101/gad.519709. 19279323 PMC2666340

[jcp70074-bib-0094] Zavodszky, E. , M. N. Seaman , K. Moreau , et al. 2014. “Mutation in VPS35 Associated With Parkinson′s Disease Impairs Wash Complex Association and Inhibits Autophagy.” Nature Communications 5, no. 1: 3828. 10.1038/ncomms4828. PMC4024763 24819384

[jcp70074-bib-0095] Zhang, Y. , B. Grant , and D. Hirsh . 2001. “RME‐8, a Conserved J‐Domain Protein, Is Required for Endocytosis in *Caenorhabditis elegans* .” Molecular Biology of the Cell 12, no. 7: 2011–2021. 10.1091/mbc.12.7.2011. 11451999 PMC55649

