# Supplementary material for: The Parkinson Disease‐Associated Mutant DNAJC13(N855S) Leads to Its Accelerated Degradation and Negatively Affects Macroautophagy and Retromer Complex‐Mediated Dynamics
Source: J Cell Physiol. 2025 Jul 27;240(7):e70074. doi: 10.1002/jcp.70074 (PMC12301596; doi:10.1002/jcp.70074)
Supplement: Supplementary file 1 — Supplemental Figure 1: HSP90‐levels are stable upon inhibition of translation independent of DNAJC13 variant expression. Supplemental Figure 2: DNAJC13 itself is not a client of autophagic degradation. Supplemental Figure 3: The mutation in DNAJC13(N855S) does not result in an overt altered subcellular localization. Supplemental Figure 4: Cathepsin D levels or cathepsin D activity in lysosomal enriched fraction are not affected by knockdown of DNAJC13. Supplemental Figure 5: Cathepsin D, LAMP2A‐level and the processing of pro‐cathepsin D remain unaffected under stable knockdown (KD) of DNAJC13 and re‐expression of wildtype DNAJC13 or DNAJC13(N855S). Supplemental Figure 6: The downregulation of the identified genes upon stable knockdown of DNAJC13 can be validated in other independent DNAJC13 knockdown cell lines. The mRNA‐expression of DNAJC13 associated genes remains largely unchanged. Table 1: Primer for RQ‐PCR. [file JCP-240-0-s001.pdf]

The Parkinson disease-associated mutant DNAJC13(N855S) leads to its accelerated degradation and negatively affects macroautophagy and retromer-complex mediated dynamics.

Anna Stein[1,§] , Stella Vo[1], Christian Freese[1], Joram Kluge[1], Joanna Maus[1], Ingrid Koziollek-Drechsler[1], Beate Silva[1], Christian Behl[1], Albrecht M Clement[1,*]

[1] Institute of Pathobiochemistry, University Medical Center of the Johannes Gutenberg-University, Duesbergweg 6, 55128, Mainz, Germany.
§ current address: Department for Hematology, Cell Therapy and Hemostaseology, University Hospital Leipzig, Liebigstr. 20, 04103 Leipzig, Germany

[*] corresponding author
Albrecht M. Clement
Duesbergweg 6, 55128 Mainz, Germany
e-mail: clement@uni-mainz.de

Supplemental Information

Supplemental Figures

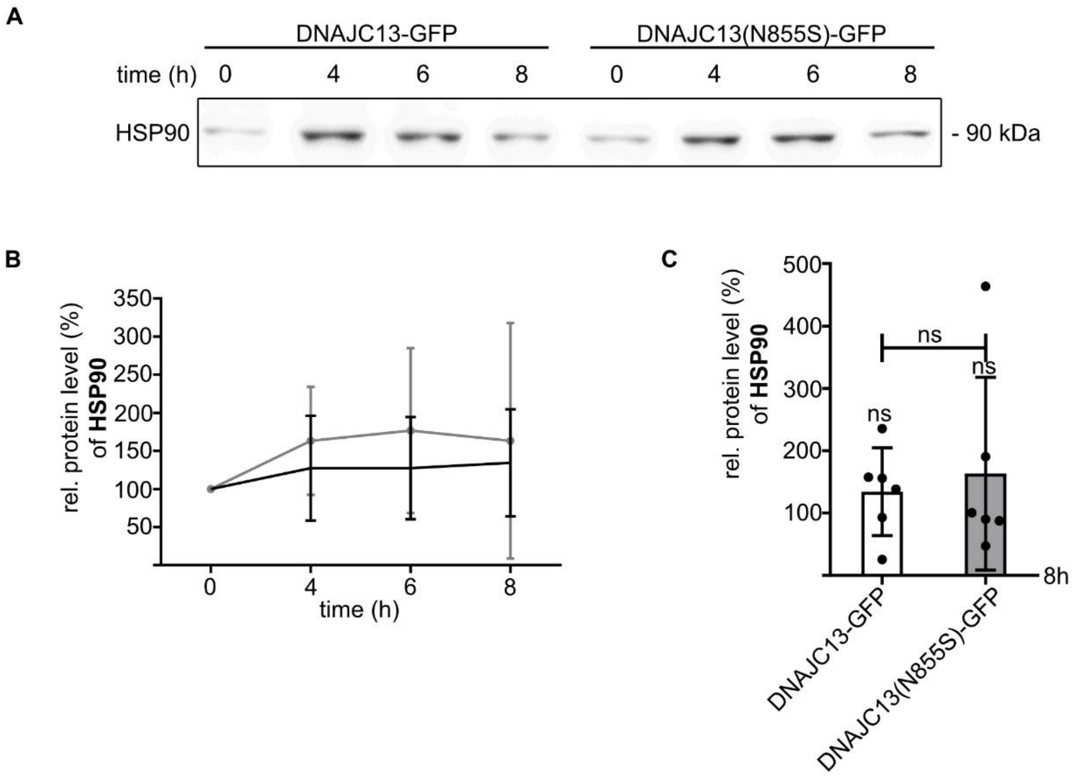

Suppl. Figure 1: HSP90-levels are stable upon inhibition of translation independent of DNAJC13 variant expression. (A) HEK293T cells were transfected with GFP-tagged DNAJC13 or DNAJC13(N855S). 24 h after transfection, protein translation was inhibited by cycloheximide for the indicated times. Equal amounts of protein were separated on an 8% SDS-PAGE gel, and after Western blotting, HSP90 was detected with a specific antibody. Band intensities were quantified and normalized to protein levels at t_0. **(B)** HSP90 protein levels remain stable throughout treatment compared to t_0. **(C)** Eight hours after cycloheximide treatment, HSP90 protein levels were not significantly reduced compared to t_0, nor was there a difference between DNAJC13-GFP and DNAJC13-(N855S)-GFP-transfection. (Mean +/- SD; n=9, t-test: *p< 0.05; **p< 0.01; ***p< 0.001)

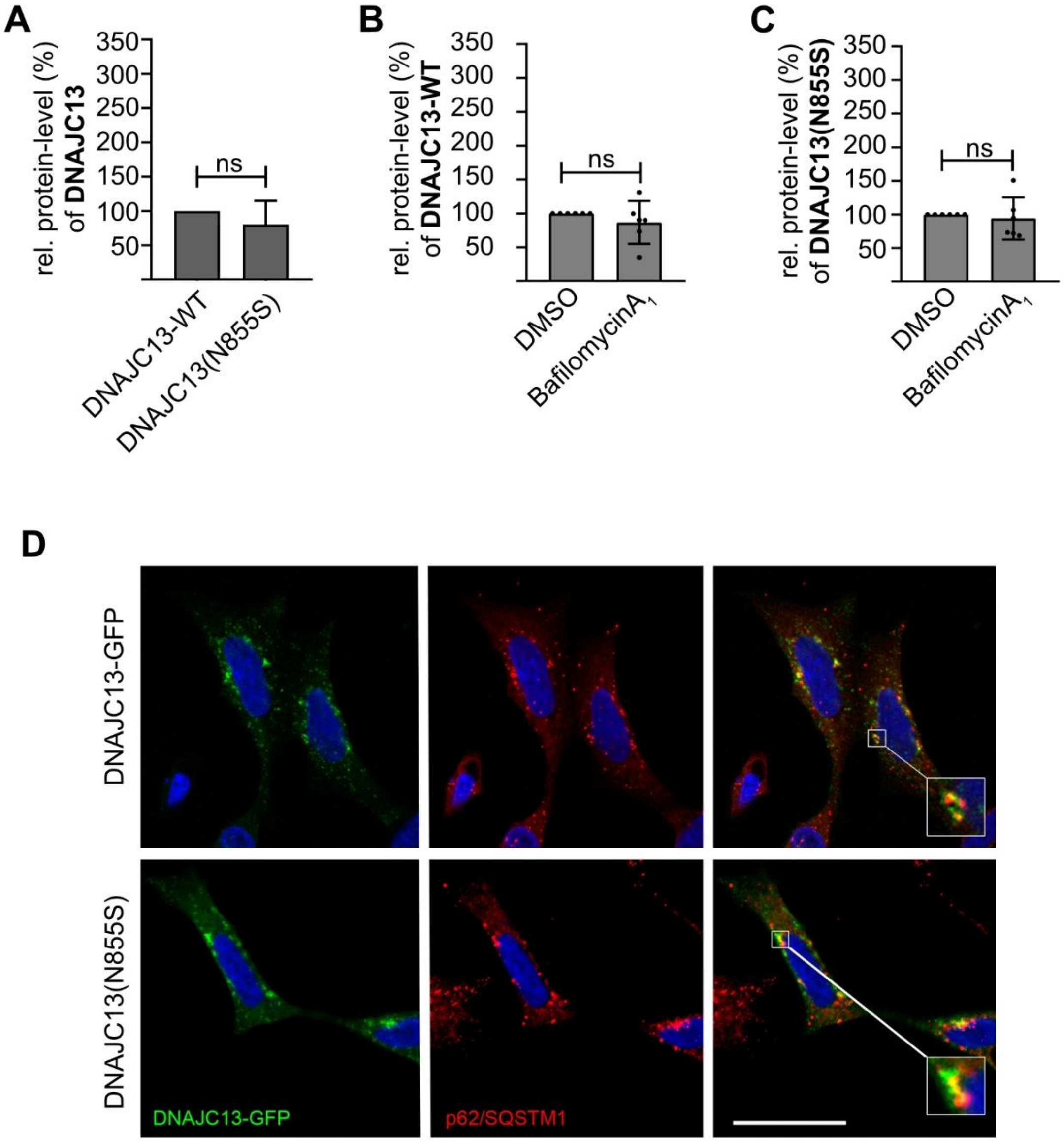

Suppl. Figure 2: DNAJC13 itself is not a client of autophagic degradation. (A) Western blot analysis of re-expressed DNAJC13(WT) and DNAJC13(N855S) in cells with stably reduced DNAJC13 levels (see Fig. 2B). GFP-tagged DNAJC13 protein levels were normalized to tubulin. (mean +/- SD; n=6; *t-test*; p=0.192). **(B)** Western blot analysis of DNAJC13 levels in stable DNAJC13 knockdown cells after re-expression of wildtype DNAJC13 and bafilomycin A_1 treatment (4 µM for 4 h) (mean +/- SD; n=6; *t-test*; p=0.347). **(C)** Western blot analysis of DNAJC13 in DNAJC13 stable knockdown cells after re-expression of mutant DNAJC13(N855S) (mean +/- SD; n=6; *t-test*; p=0.668). The lack of accumulating DNAJC13 protein upon bafilomycin A_1 treatment suggests that DNAJC13 is not a primary target of autophagic degradation. **(D)** The mutation in DNAJC13(N855S) does not result in an overt altered subcellular localization towards autophagosomes. HEK293A-cells were transiently transfected with DNAJC13(WT)-GFP or DNAJC13(N855S)-GFP and treated with bafilomycin A_1 for 4 h (4 µM). Cells were fixed and stained for p62/SQSTM1 with specific primary and

corresponding fluorescent-tagged secondary antibodies. The green signal in confocal images indicates the transfected GFP-tagged protein. The red signal shows p62/-SQSTM1. (scale bar: 20 µm).

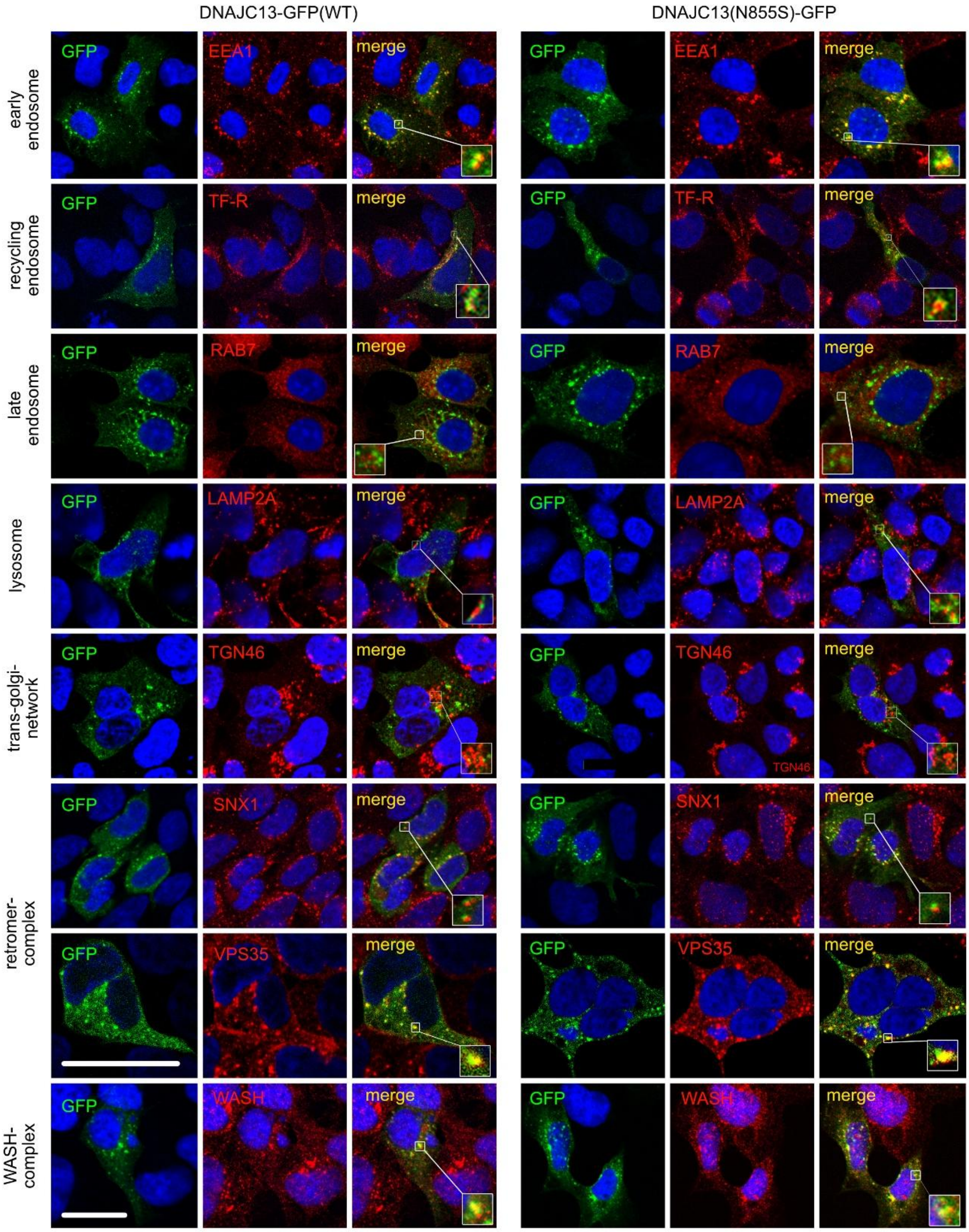

Suppl. Figure 3: The mutation in DNAJC13(N855S) does not result in an overt altered subcellular localization. HEK293A-cells were transiently transfected with DNAJC13-GFP or DNAJC13(N855S)-GFP. Cells were fixed and stained for endosomal compartments, the retromer, and WASH complexes with specific primary and corresponding fluorescent-tagged secondary antibodies. The green signal of the confocal images indicates the transfected GFP-tagged protein. The red signal indicates particular marker proteins for each endosomal compartment. The colocalization of GFP-tagged DNAJC13 and DNAJC13(N855S) with

endosomal compartments, the retromer, and WASH complexes show no obvious differences (scale bar: 20 µm).

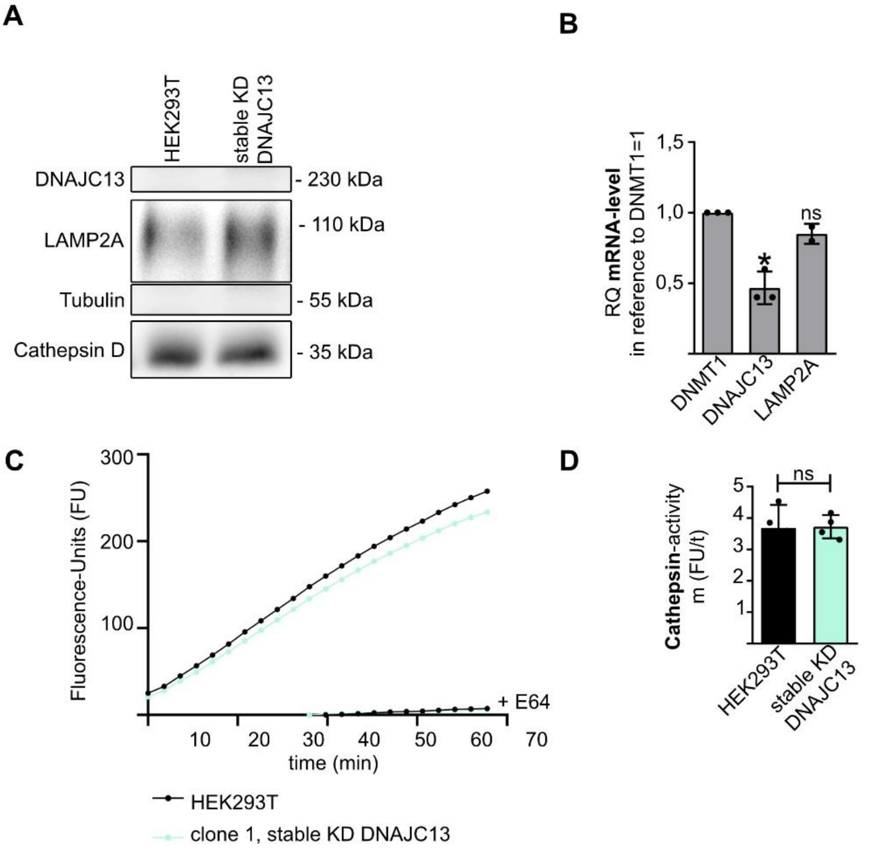

Suppl. Figure 4: Cathepsin D levels or cathepsin D activity in lysosomal enriched fraction are not affected by knockdown of DNAJC13. **(A)** Lysosome-enriched fractions were characterized by Western blot analysis showing cathepsin D and LAMP2A. DNAJC13 and the cytosolic protein tubulin were not detected in these fractions. **(B)** Analysis of mRNA-expression by RT-qPCR showing significant knockdown of DNAJC13 and unchanged expression of LAMP2A upon stable knockdown of DNAJC13. DNA-methyltransferase 1 (DNMT1) served as a reference (n=3, mean +/- SD, t-test; *p< 0.05;**p< 0.01;***p< 0.001). **(C)** Lysosome-enriched fractions were incubated with a fluorogenic substrate for different cathepsins, like cysteine proteases cathepsin B, C, K, and L. . The increase of fluorescence over time (slope of the graph) is considered as the activity of the enzyme. Shown is one representative experiment. E-64-D is a specific inhibitor of cysteine proteases and serves as a negative control. The slopes of one experiment were calculated from a technical triplicate. The enzymatic activity of cathepsin is independent of DNAJC13 protein levels. (Mean +/- SD; n= 4, *t-test*: *p< 0.05; ns not significant)

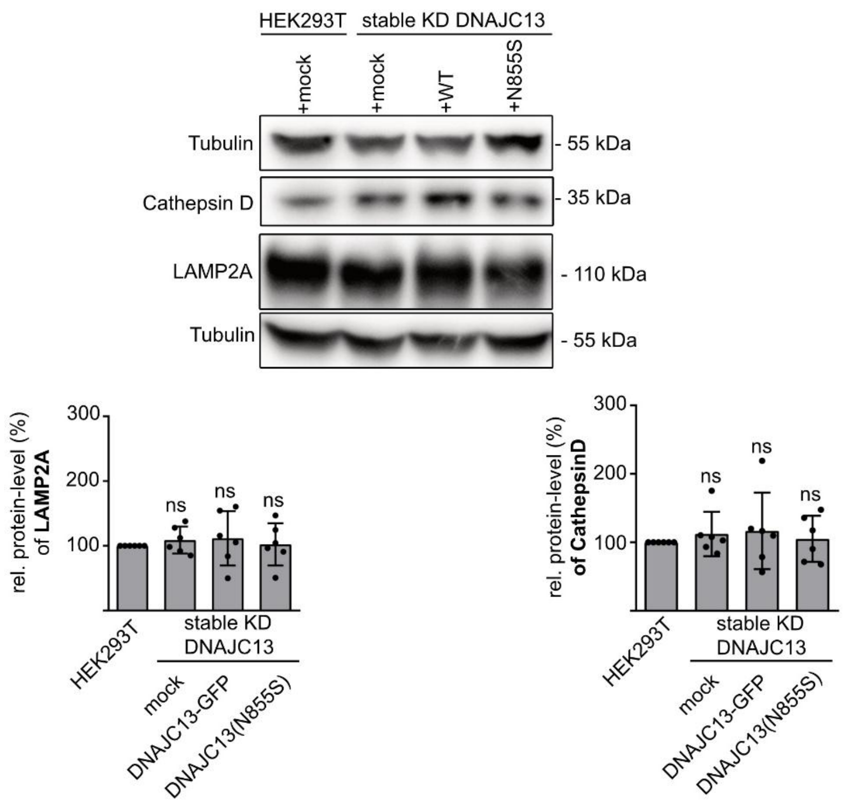

Suppl. Figure 5: Cathepsin D, LAMP2A-level and the processing of pro-cathepsin D remain unaffected under stable knockdown (KD) of DNAJC13 and re-expression of wildtype DNAJC13 or DNAJC13(N855S). (A) Western blot analysis of total lysates of wildtype cells and stable DNAJC13 knockdown cells upon the transient transfection with a mock vector, wildtype DNAJC13 or DNAJC13(N855S). **(B)** Quantification of Western blots in A: The ratio of cathepsin D / pro-cathepsin D is stable under all conditions (n=4, mean +/- SD, one-way-ANOVA: *p<0.05, ns not significant). **(C,D)** LAMP2A (n=5) (C) and cathepsin D (n=6) (D) protein levels were determined and normalized to tubulin. The protein level of both proteins is independent of DANJC13 levels, even after re-expression of (mean +/- SD, One-way-ANOVA: *p< 0.05, ns not significant).

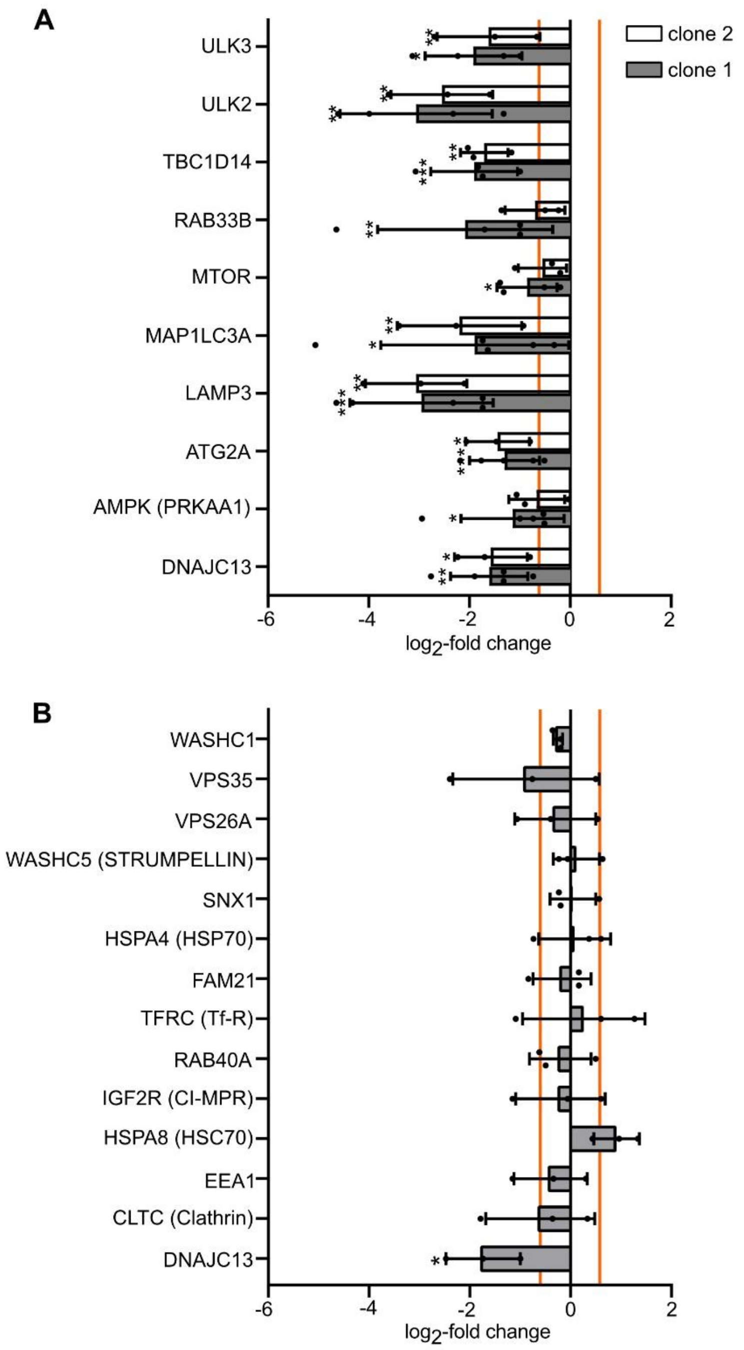

Suppl. Figure 6: The downregulation of the identified genes upon stable knockdown of DNAJC13 can be validated in other independent DNAJC13 knockdown cell lines. The mRNA-expression of DNAJC13 associated genes remains largely unchanged. (A) RT-qPCR-analysis upon stable knockdown of DNAJC13 in two independent DNAJC13 knockdown cell lines (mean +/- SD; n=4; *t-test*: *p< 0.05; **p< 0.01; ***p< 0.001). **(B)** mRNA-expression of DNAJC13 associated genes. The mRNA-expression of DNAJC13 is significantly reduced (mean +/- SD; n=4; *t-test*: *p< 0.05). RQ of 0.6 (log_2=-0.6) has been considered a downregulation. RQ of 1.5 (log_2= 0.58) has been considered an upregulation, both indicated by the red line.

Supplemental Table

Table 1: Primer for RQ-PCR

target mRNA	sequence
alsin for	5´-CCTTGACAGGCAGGAAGAAG-3´
alsin rev	5´-CTGCTTGGGTGTCAGGGTAT-3´
ampk/PRKAA1 for	5´-CCTCAAGCTTTCAGGCATC-3`
ampk/PRKAA1 rev	5´-TTTCATCCAGCCTTCCATTC-3`
ATG-2A for	5´-AAGTACTTCCTGCCCCAGGT-3`
ATG-2A rev	5´-TCTCCAGGGATCACCATCT-3`
ATG-2B for	5´-AGCTGGCATGAGCAGATTTT-3`
ATG-2B rev	5´-GTGCACAGCTCCAAAGATGA-3`
ATG-3 for	5`-GATGGCGGATGGGTAGATACA-3`
ATG-3 rev	5`-TCTTCACATAGTGCTGAGCAATC-3`
ATG-4B for	5`-ATGGACGCAGCTACTCTGAC-3`
ATG-4B rev	5`-TTTTCTACCCAGTATCCAAACGG-3`
ATG-4C for	5´-GTTGAAGAAGCAAGGCATCC-3`
ATG-4C rev	5´-TCGGTGTTGGTTCTTTCTCC-3`
ATG-4D for	5´-AAGGCAGGTGACTGGTATGG-3`
ATG-4D rev	5´-ACAGACTTCCACTCGGCTGT-3`
ATG-5 for	5´-TTCGAGATGTGTGGTTTGGA-3`
ATG-5 rev	5´-ATGGTTCTGCTTCCCTTTCA-3`
ATG-7 for	5`-TGGAACAAGCAGCAAATGAG-3`
ATG-7 rev	5`-AGACAGAGGGCAGGATAGCA-3`
ATG-9A for	5´-GTCAGCTGCGTGGACTATGA-3`
ATG-9A rev	5´-GACTTGAGCAGGCAAAAAGG-3`
ATG-12 for	5`-TAGAGCGAACACGAACCATCC-3`
ATG-12 rev	5`-CACTGCCAAAACACTCATAGAGA-3`
ATG-14 for	5´-AGCAGACCTTGAGGAGTCCA-3`
ATG-14 rev	5´-CCACAGACTGGGAAGGGATA-3`
ATG-16L1 for	5`-ATGCGCGGATTGTCTCAGG-3`
ATG-16L1 rev	5`-GTCCACTCATTACACATTGCTCT-3`
ATG-16L2 for	5´-TCTGAGGTCAATGCTGTTCG-3`
ATG-16L2 rev	5´-AGGGGTCAAAGTCCACACTG-3`
b2m for	5´-GTGCTCGCGCTACTCTCTCT-3`
b2m rev	5´-TCTCTGCTGGATGACGTGAG-3`
beclin1 for	5´-AGGTTGAGAAAGGCGAGACA-3`
beclin1 rev	5´-GCTTTTGTCCACTGCTCCTC-3`
clathrin for	5´-ACTTAGCCGGTGCTGAAGAA-3´
clathrin rev	5´-AACCGACGGATAGTGTCTGG-3´
dnmt1 for	5´-ACAGTACCAGCCCATCCTCA-3´
dnmt1 rev	5´-AGGCTTTGCCGGCTTCC-3´
eea1 for	5´-TGCATCTGAAACCTCACTGC-3´
eea1 rev	5´-CCATGCTGCTCCTTTTCTTC-3´
fam21 for	5´-ACAGGCAAAGTCCAATCCAC-3´
fam21 rev	5´-TGACTCACAGTGGCTTCTGG-3´
gabarap for	5`-AGAAGAGCATCCGTTCGAGA-3`
gabarap rev	5`-GAGCTTTGGGAGCCTTTTCT-3`
gabarapL1 for	5´-GGTCCCCGTGATTGTAGAGA-3`
gabarapL1 rev	5´-GGAGGGATGGTGTTGTTGAC-3`
gabarapL2 for	5´-AAATATCCCGACAGGGTTCC-3`
gabarapL2 rev	5´-CAGGAAGATCGCCTTTTCAG-3`

hsc70 for	5´-GGAGGTGGCACTTTTGATGT-3
hsc70 rev	5´-AGCAGTACGGAGGCGTCTTA-3
hsp70 for	5´-CAAGATCACCATCACCAACG-3´
hsp70 rev	5´-TCGTCCTCCGCTTTGTACTT-3´
lamp1 for	5´-CTGCCTTTAAAGCTGCCAAC-3`
lamp1 rev	5´-TGTTCTCGTCCAGCAGACAC-3`
lamp2 for	5´-AATGCCACTTGCCTTTATGC-3`
lamp2 rev	5´-CAGTGCCATGGTCTGAAATG-3`
lamp3 for	5´-CCCCACCAGCTCATACAACT-3`
lamp3 rev	5´-CTGGAAGGGTGGTCTGGTTA-3`
lrrk1 for	5´-CCTGCCCTTTGAAATGTTGT-3´
lrrk1 rev	5´-AGCTGGTTCCCCTGTAACCT-3´
m6pr for	5´- ACTCCTTGCTTAGCGGACAA-3´
m6pr rev	5´-TCGCTGGTCCTTAACAGCTT-3´
map1lc-3A for	5´-CATGAGCGAGTTGGTCAAGA-3`
map1lc-3A rev	5´-CTCGTCTTCTCCTGCTCGT-3`
map1lc-3B for	5`-CTGTTGGTGAACGGACACAG-3`
map1lc-3B rev	5`-CTGGGAGGCATAGACCATGT-3`
map1lc-3C for	5´-CGGAAGCCTTTTACTTGCTG-3`
map1lc-3C rev	5´-GTCTGTCCTCAAGGCTGCTC-3`
mtor for	5´-CCTGCCACTGAGAGATGACA-3`
mtor rev	5´-TCCGGCTGCTGTAGCTTATT-3`
nbr-1 for	5`-GTTGCTGCCTCTGCATACAA-3`
nbr-1 rev	5`-TTTCTTCAGCAGCCGTAGGT-3`
ndp-52/calcoco2 for	5`-GTGGAAGACAACCCGTGAGT-3`
ndp-52/calcoco2 rev	5`-TTCTGGACGGAATTGGAAAG-3`
p62/SQSTM-1 for	5´-GTGGTAGGAACCCGCTACAA-3`
p62/SQSTM-1 rev	5´-GAGAAGCCCTCAGACAGGTG-3`
rab5 for	5´-TTGCCAAAGAATGAACCACA-3´
rab5 rev	5´-GGTTGTGTGGGTTCGGTAAG-3´
rab7A for	5´- CTGACCAAAGGAGGTGATGGT-3`
rab7A rev	5´-GTAGAAGGCCACACCGAGAG-3`
rab11A for	5´-AGCAAGAGCACCATTGGAGT-3`
rab11A rev	5´-CCCTGCTGTGTCCCATATCT-3`
rab18 for	5´-CTGAAATTTGCACGAAAGCA-3`
rab18 rev	5´-CACAGTCCAGGGGTCTGAAT-3`
rab3gap-1for	5´-CTGACCGAAGGGATCATTGT-3`
rab3gap-1 rev	5´-CCTTTCGACGGCAAATTTTA-3`
rab3gap-2 for	5´-TGAGAATGGTGTGCTCTTGC-3`
rab3gap-2 rev	5´-TGATTTCGACAAGCACGAAG-3`
rab24 for	5´-GCAAGACTAGCCTGGTGGAG-3´
rab24 rev	5´-CTTGGCACCCCGATAGTAGA-3´
rab26 for	5´-AAAGTTCTGGACGTGGATGG-3´
rab26 rev	5´-GGTGACATCGTAGAGCAGCA-3´
rab33b for	5`-GCTGAGGAGATGGAGTCGTC-3´
rab33b rev	5´-GAGTCGCCGATCACGATTAT-3´
rab40a for	5´-GAGCTTGCAAGACCTCTGCT-3´
rab40a rev	5´-TCTTGTGAGTGGAGCTGGTG-3´
dnajc13_RT-1 for	5´-GGGATAGGATGGGCATTGCT-3´
dnajc13_RT-1 rev	5´-GCATGGTCTTGACCAAACCTATAA-3´
rpl-19 for	5`-GAAATCGCCAATGCCAACTC-3`
rpl-19 rev	5`-TTCCTTGGTCTTAGACCTGCG-3`
snx1 for	5´-ATTGAGTGGTGCTGGTCTCC-3´
snx1 rev	5´-ACCTCCTGGAGCTTCTCCTC-3´

strump for	5´-AAACGACTTTGGTTGGCATC-3´
strump rev	5´-GTCGCTCCCAACTCTTTCAG-3´
tbc1d5 for	5´-GCCCAGTGAGGAAATGAAAA-3`
tbc1d5 rev	5`-CTTTCCCCTTCTGACCATCA-3´
tbc1d14 for	5´-GGGAAACAATGTGGTGCTCT-3´
tbc1d14 rev	5`-TCCACTTCAGAGCCTCCTGT-3´
tbc1d20 for	5´-CACCAGGCTCTGAACAGTGA-3`
tbc1d20 rev	5´-CATTGGCATTGACATTGAGG-3`
tmem230 for	5´-CACTTGCCACTGTGCTGTTT-3´
tmem230 rev	5´-AAAATCCGGGTAGGAACACC-3´
transferrin-R for	5´-AAAATCCGGTGTAGGCACAG-3´
transferrin- R rev	5´-CACCAACCGATCCAAAGTCT-3´
trappc3 for	5´-TGAGCTCTTCACCCTGACCT-3´
trappc3 rev	5´-CCCATTTTGTCCAGCTGTTT-3´
ulk1 for	5´-CAGAACTACCAGCGCATTGA-3`
ulk1 rev	5´-TCCACCCAGAGACATCTTCC-3`
ulk2 for	5´-TTTGGTGCCACACAACATCT-3`
ulk2 rev	5´-GGAACTGGAATTGGTGCTGT-3`
ulk3 for	5´-CAGGATTCTGCCTGAGAAGG-3`
ulk3 rev	5´-ATGTGTTGTGCGAAACCAAA-3`
uvrag for	5´-GCACCCTAGCCAAGAACAAG-3`
uvrag rev	5´-AGCTTCTGAGACTGGGTGGA-3`
vapa for	5´-TGAGCAGCATCAACAACACA-3`
vapa rev	5´-TGGTTTTGGCATAGGTCCAT-3`
vapb for	5´-TGTTTGAATTGCCAGCAGAG-3`
vapb rev	5´-GCCTCTGAACTTCACCTTGC-3`
VPS26 for	5´-CCAATGTCCGCTTGAGGTAT-3´
VPS26 rev	5´-TGGCAAGCTGGTGAACAATA-3´
VPS35 for	5´-TTCACAGCAGGTGGCTACAG-3´
VPS35 rev	5´-TCCACACGATCAGGGTAACA-3´
wash1 for	5´-ATCTCCCAGCAGGTAGAGCA-3´
wash1 rev	5´-TTCTTGCTGCCCTTGATCTT-3´
wipi1 for	5´-CCAGACCCAGTGCATCTTCA-3`
wipi1 rev	5´-TCATGACTGCTTCGTTTTGC-3`
wipi2 for	5´-CTCTCACGACTGCCCCTTAG-3`
wipi2 rev	5´-ACCCAGGTCGTCTGTGTAGG-3`
